# Mouse entorhinal cortex encodes a diverse repertoire of self-motion signals

**DOI:** 10.1038/s41467-021-20936-8

**Published:** 2021-01-28

**Authors:** Caitlin S. Mallory, Kiah Hardcastle, Malcolm G. Campbell, Alexander Attinger, Isabel I. C. Low, Jennifer L. Raymond, Lisa M. Giocomo

**Affiliations:** Department of Neurobiology, Stanford University School of Medicine, Stanford, CA USA

**Keywords:** Neuroscience, Neural circuits

## Abstract

Neural circuits generate representations of the external world from multiple information streams. The navigation system provides an exceptional lens through which we may gain insights about how such computations are implemented. Neural circuits in the medial temporal lobe construct a map-like representation of space that supports navigation. This computation integrates multiple sensory cues, and, in addition, is thought to require cues related to the individual’s movement through the environment. Here, we identify multiple self-motion signals, related to the position and velocity of the head and eyes, encoded by neurons in a key node of the navigation circuitry of mice, the medial entorhinal cortex (MEC). The representation of these signals is highly integrated with other cues in individual neurons. Such information could be used to compute the allocentric location of landmarks from visual cues and to generate internal representations of space.

## Abstract (web summary)

Here, the authors show that mouse medial entorhinal cortex encodes three-dimensional head movement as well as eye position and velocity. These self-motion signals are represented conjunctively in individual neurons alongside body position, running speed, and azimuthal head direction.

## Introduction

Vision is a primary sensory modality for navigation^1,2^, with visual input providing information critical to the accuracy of each of the three main classes of navigational strategies, map-based navigation, route-based navigation, and path-integration^3^. During route-based and map-based navigation, position in the environment is calculated based on familiar landmarks, and visual features act as points of reference. During path-integration-based navigation, position relative to a starting point is estimated by continuously tracking self-motion cues, with optic flow providing one self-motion cue, and familiar visual features providing error correction signals^4,5^. Accurate spatial interpretation of visual cues requires that the position and motion of the retina be taken into account. More specifically, to estimate the position of visual features in the world, the direction that the retina is pointing must be considered, which is influenced by the position of the eye within the orbit as well as the position and orientation of the head and body within the environment. Likewise, estimation of self-motion relative to landmarks from optic flow signals requires consideration of eye and head velocity. Recent reports have quantified considerable motion of not only the head but also the eyes during active navigation in rodents^6–8^, bringing to the forefront questions of whether and how these movements are represented in the neural circuitry supporting navigation.

“Head direction” cells, whose firing encodes head position in the azimuth plane^9–14^, have been well-documented in multiple brain regions and species, however only a few studies have analyzed coding of other aspects of head and eye movements in brain regions associated with navigation. Neurons in the presubiculum of bats, and the thalamus and retrosplenial cortex of rodents, encode head movements not just in the azimuthal plane, but in the pitch and roll planes as well^14,15^. In primates, neurons encoding eye position have been reported in the medial entorhinal cortex (MEC), a key node of the brain’s navigation circuitry^16,17^. A subset of primate MEC neurons modulates their activity with the animal’s gaze in a periodic manner similar to the representation of allocentric body position by rodent entorhinal grid cells^18^, raising the possibility that eye position coding in the MEC enables primates to explore a visual scene with saccadic eye movements, functioning like body position coding in rodents. Determining whether eye position signals are present in the MEC of other species may elucidate computations performed by the MEC across species, as well as reveal species-specific specializations.

Here, we investigate whether the position and velocity of the head and eyes are represented in rodent MEC. MEC contains a constellation of functionally defined cell types that encode an animal’s location in allocentric space^10,19–23^, including grid cells that fire in periodic spatial locations, border cells that are maximally active near environmental boundaries, object-vector cells that fire at specific locations relative to objects, and cells with stable but non-geometric spatial firing patterns^10,19–22^. Visual input directly influences at least a subset of these representations, as the firing patterns of grid and border cells are locked to visual landmarks^22,23^, can be elicited by visual stimuli alone^24^, and degrade in complete darkness^23,25^. We report that MEC neurons encode multiple parameters of head and eye movements, which could support the integration of visual features into an allocentric representation of space. Using two experimental set-ups in which we either track the 3D position and velocity of the head during random foraging, or the position and velocity of the eye during head-fixed navigation, we identified neural activity in the MEC associated with the pitch and roll position and angular azimuthal velocity of the head, as well as position and velocity of the eyes. These signals are jointly represented by single neurons along with other navigationally relevant variables.

## Results

### Entorhinal neurons encode head movements in three dimensions

**Fig. 1:**
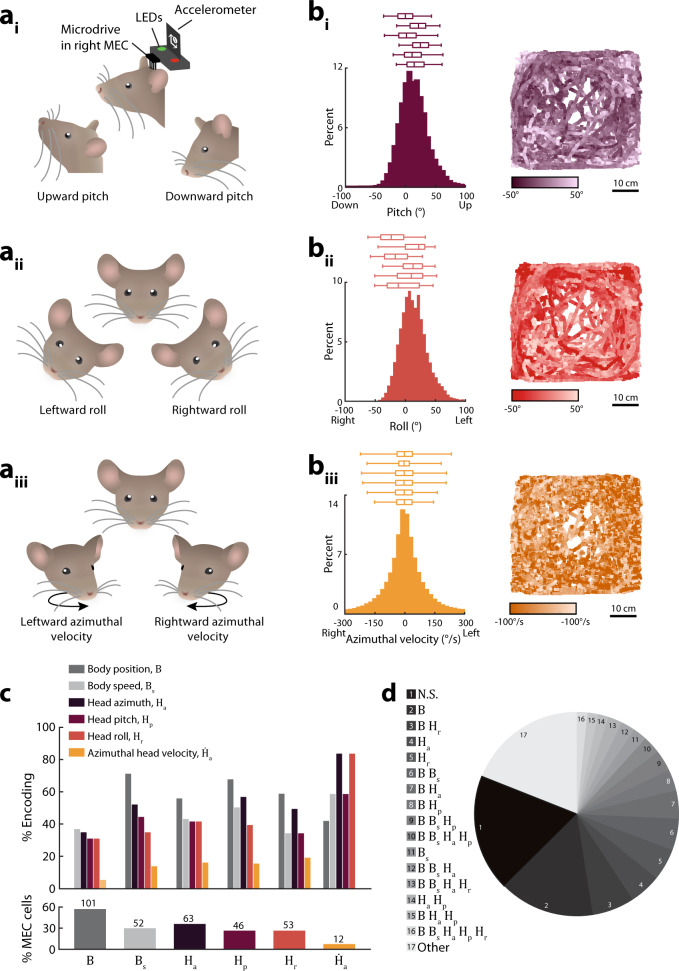
MEC neurons encode three Euler-angles of head position. **a** During open field exploration, head pitch (**a**_**i**_) and roll (**a**_**ii**_) were measured with an accelerometer affixed to the headstage^7,26,62^; angular head velocity in the azimuthal (yaw) plane (**a**_**iii**_), azimuthal head direction, body position, and linear body speed (not pictured) were measured from two LEDs affixed to the headstage^9,10^. **b** Histograms show behavioral distributions in an example session. Plots on right show behavior in the recording arena. Boxplots show distributions observed in individual mice, averaged across sessions. Box, interquartile range; vertical line, median; whiskers extend from the 2.5th to 97.5th percentiles. Mean ± standard error of mean [sem] across mice; pitch: 2.5th percentile = −23.0 ± 4.5°, 25th percentile = −1.4 ± 4.1°, median = 11.5 ± 4.1°, 75th percentile = 25.8 ± 6.0°, 97.5th percentile = 55.6 ± 4.1° (*n* = 6 mice); roll: 2.5th percentile = −50.9 ± 3.6°, 25th percentile = −20.3 ± 7.0°, median = −0.8 ± 7.6°, 75th percentile = 19.3 ± 6.0°, 97.5th percentile = 43.6 ± 4.0° (*n* = 6 mice); azimuthal head velocity: 2.5th percentile = −389.9 ± 34.7°/s, 25th percentile = −37.7 ± 3.9°/s, median = −0.1 ± 0.3°/s, 75th percentile = 36.4 ± 3.8°/s, 97.5th percentile = 385.1 ± 34.7°/s (*n* = 25 mice from two datasets; Methods). **c** Bottom: Bar graph showing the percentage of cells significantly encoding each variable, with the cell count at top. Top: Bar graph showing the percentage of cells jointly encoding all combinations of two variables. *B*, body position; *B*_*s*_, body speed; *H*_*a*_, azimuth head direction; *H*_*p*_, pitch; *H*_*r*_, roll; $\dot H_a$, azimuthal head velocity. **d** Pie chart illustrating tuning to multiple variables (*n* = 179 cells). Combinations observed in fewer than three cells are grouped together [“other”]. N.S., no significant tuning to any variable. Few cells encoded body speed, azimuthal head direction, pitch, roll, or azimuthal head velocity alone (% of MEC cells non-conjunctively encoding: body speed, 2%; azimuthal head direction, 5%; pitch, 1%; roll, 5%, azimuthal head velocity, 0%). In contrast, 15% of MEC cells encoded body position non-conjunctively.

During navigation, rodents move their heads about the three Euler axes of azimuth (yaw), pitch, and roll^7,26^. We used an accelerometer and LEDs affixed to the microdrive headstage to monitor 3D head position and angular velocity of freely foraging mice while recording neural activity from the right MEC using tetrodes (Fig. 1a, b; Supplementary Fig. 1). The animal’s body position and speed were also tracked via headstage LEDs. To assess how MEC neural activity varies with 3D head movement alongside previously recognized navigational variables, we fit a series of linear-nonlinear (LN) Poisson models to the spiketrain of each cell^27^ (Methods, Supplementary Fig. 2). The model included variables for 3D head position (azimuth [*H*_*a*_], pitch [*H*_*p*_], roll [*H*_*r*_]) and angular velocity (azimuthal velocity [$\dot H_a$], pitch velocity [$\dot H_p$], roll velocity [$\dot H_r$]), along with body position [*B*] and linear body speed [*B*_*s*_]. This method has effectively characterized conjunctive coding of other navigation-related variables in the MEC^27^ and yields low rates of false detection (Supplementary Figs. 3, 4).

Consistent with previous work, a large fraction of the 179 MEC neurons recorded encoded azimuthal head direction (*n* = 63 cells, 35%)^10^. In addition, 46 neurons [26%] encoded head pitch position and 53 [30%] encoded head roll position. Few cells encoded angular velocity along the pitch or roll axes ($\dot H_p$ *n* = 4/179, and $\dot H_r$ *n* = 5/179) and were not examined further. A small population of neurons encoded azimuthal head velocity ($\dot H_a$ *n* = 12/179 cells; 7%). Tuning of neurons to azimuthal head velocity was verified in an additional dataset in which head movements were only measured in the azimuthal plane (*n* = 55/1021 cells, 5%; Methods). Individual neurons commonly encoded information about multiple variables corresponding to head or body movement (95/179 cells encoded > 1 variable), consistent with prior reports establishing the ubiquity of conjunctive coding within MEC^10,19,27^ (Fig. 1c, d).

**Fig. 2:**
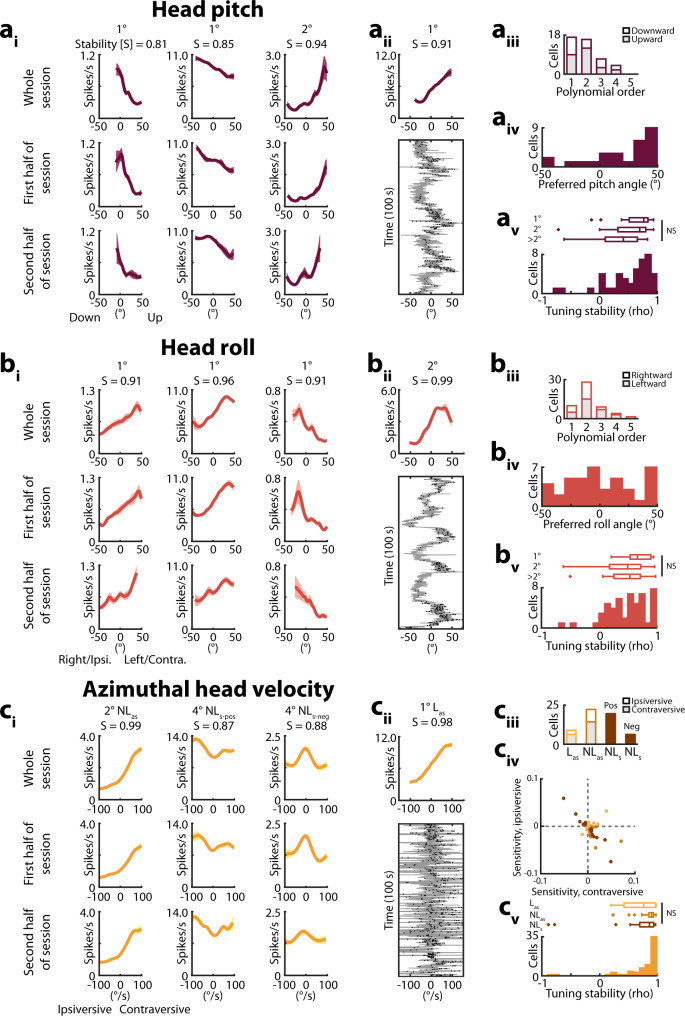
Heterogenous tuning to pitch, roll, and azimuthal head velocity. **a** Model-derived tuning curves (mean ± sem of 10 models) for cells significantly encoding pitch (Supplementary Figs 5–8). The stability (“S”) and tuning curve class (1° = linear, etc.) are indicated at the top. **a**_**ii**_ Bottom: Spiking from a pitch-encoding cell. Gray trace, pitch position. Black dots, spikes. Top: The associated tuning curve. **a**_**iii**_ Summary of tuning curve classes. Bar color denotes downward/upward preference. **a**_**iv**_ Preferred position for cells non-linearly encoding pitch. **a**_**v**_ Stability of tuning to pitch. Boxplots: stability did not significantly differ between classes (median [1st–3rd quartile]; 1° = 0.77 [0.52–0.84], *n* = 17; 2° = 0.70 [0.32–0.80], *n* = 16; >2° = 0.41 [0.10–0.66], *n* = 11; *Χ*^*2*^ = 4.21, *P* = 0.12, df = 2; Kruskal–Wallis test). **b**_**i**_ Tuning curves for cells significantly encoding roll. **b**_**ii**_ Spiking from a roll-encoding cell. **b**_**iii**_ Summary of tuning curve classes. Bar color denotes directional preference. **b**_**iv**_ Preferred position for cells non-linearly encoding roll. **b**_**v**_ Stability of tuning to roll. Boxplots: stability did not significantly differ between classes (median [1st–3rd quartile]; 1° = 0.68 [0_._46–0.91], *n* = 10; 2° = 0.54 [0.19–0.76], *n* = 28; >2° = 0.37 [0.26–0.71], *n* = 14; *Χ*^*2*^ = 2.7, *P* = 0.26, df = 2; Kruskal–Wallis test). **c**_**i**_ Tuning curves for cells significantly encoding azimuthal head velocity (*n* = 67 cells from two datasets; Methods). L_as_, linear asymmetric; NL_as_, nonlinear-asymmetric; NL_s_, nonlinear-symmetric; Pos, positive; Neg, negative. **c**_**ii**_ Spiking from an azimuthal head velocity-encoding cell. **c**_**iii**_ Summary of tuning curve classes. Bar color indicates directional preference. **c**_**iv**_ Cells' sensitivity in the contraversive versus ipsiversive rotational ranges. Units = spikes/s/°/s. **c**_**v**_ Stability of tuning to azimuthal head velocity. Boxplots, stability did not significantly differ between classes (median [1st–3rd quartile]; L_as_ = 0.78 [0.44–0.99], *n* = 10; NL_as_ = 0.92 [0.87–0.96], *n* = 28; NL_s_ = 0.88 [0.71–0.96], *n* = 26; *Χ*^*2*^ = 0.73, *P* = 0.70, df = 2; Kruskal–Wallis test). Boxplots; box, interquartile range; solid line, median; whiskers, range; outliers plotted separately. NS; not significant.

The relationship between the firing activity of MEC neurons, pitch, and roll was further characterized by quantifying the shapes of the model-derived tuning curves for neurons significantly modulated by these variables (Fig. 2a, b, Supplementary Figs. 5–8). We used an unbiased approach to characterize the shape of these tuning curves by fitting a series of polynomial functions (up to 5th order) to each tuning curve with sufficient behavioral coverage, and identifying the lowest-order polynomial that explained at least 90% of the variance (Fig. 2a_iii_, b_iii_, Supplementary Figs. 7, 8; Methods). Nearly all pitch and roll tuning curves were well-fit (>90% variance explained) by a 5th or lower order polynomial (*H*_*p*_ *n* = 44/46, *H*_*r*_ *n* = 52/53 cells). Both pitch-encoding and roll-encoding cells tended to be well-fit by simpler polynomials (Fig. 2a_iii_, b_iii_). The majority of cells encoding pitch exhibited either linear or quadratic relationships between firing rate and pitch position (linear *n* = 17/46, quadratic *n* = 16/46 cells, Fig. 2a_iii_), while the majority of cells encoding roll exhibited quadratic relationships between firing rate and roll position (linear *n* = 10/53, quadratic *n* = 28/53 cells, Fig. 2b_iii_). Similar proportions of pitch-encoding cells were maximally activated by upward or downward tilts of the head (upward-preferring *n* = 26/44 cells, *Z* = 1.06, *P* = 0.29, binomial test; Fig. 2a_iii,iv_), and similar proportions of roll-encoding cells preferred leftward (contraversive) and rightward (ipsiversive) tilts (leftward-preferring *n* = 30/52 cells, *Z* = 0.82, *P* = 0.41; Fig. 2b_iii,iv_). The stability of tuning to pitch or roll, quantified as the Pearson’s correlation coefficient between tuning curves generated for the first and second halves of the session, was comparable to that of azimuthal head direction (median [1st–3rd quartile]; pitch = 0.67 [0.35–0.80], *n* = 44; roll = 0.54 [0.23–0.74], *n* = 52; azimuthal head direction = 0.65 [0.47–0.81], *n* = 63; *Χ*^*2*^ = 4.26, *P* = 0.12, df = 2, Kruskal–Wallis test; Fig. 2a_v_, b_v_).

A similar approach was used to characterize the tuning curves of MEC cells encoding azimuthal head velocity ($\dot H_a$; *n* = 64/67 cells well-fit by a 5th or lower order polynomial, combined datasets from right and left MEC; Fig. 2c_i-ii_). A subpopulation of neurons linearly increased or decreased their firing with azimuthal head velocity (Fig. 2; *n* = 10/67 cells, termed linear-asymmetric, L_as_, following^28,29^). However, most tuning curves were best fit by higher order polynomials (quadratic *n* = 30/67, cubic *n* = 8/67, quartic *n* = 16/67 cells). Maxima and minima of these curves clustered near 0°/s (Supplementary Fig. 8). By comparing the slope below and above 0°/s (i.e., for ipsiversive versus contraversive rotations of the head, Fig. 2c_iii_), we identified two classes of nonlinear cells (Fig. 2c_iv_). Half of $\dot H_a$ cells increased or decreased their firing rates with azimuthal head speed in only one direction, with little or no change in activity for head rotation in the opposite direction (nonlinear-asymmetric [NL_as_], terminology following^28,29^, *n* = 28/64 cells). The other half of cells equally modulated their firing rates with head speed regardless of direction (nonlinear-symmetric [NL_s_], terminology following^28,29^, *n* = 26/64 cells)^28,29^. Whereas the majority of symmetric cells increased their firing rates with increasing head speeds [NL_s-pos_] (*n* = 20/26), others fired maximally when the head was stationary [NL_s-neg_] (*n* = 6/26, *Z* = 2.55, *P* = 0.009). Similar proportions of asymmetric cells preferred ipsiversive (i.e., head rotation toward the side of recording) versus contraversive head movements (contraversive-preferring *n* = 23/38, *Z* = 1.16, *P* = 0.26). Tuning to azimuthal head velocity was highly stable across the first and second half of the recording session (median [1st–3rd quartile]; 0.91 [0.74–0.96], *n* = 64; Fig. 2c_v_), and comparable to the stability of tuning to body speed (0.86 [0.63–0.95], *n* = 52; *Z* = 1.66, *P* = 0.097, Wilcoxon rank-sum test).

### Conjunctive coding of head movements and other navigation-related variables

**Fig. 3:**
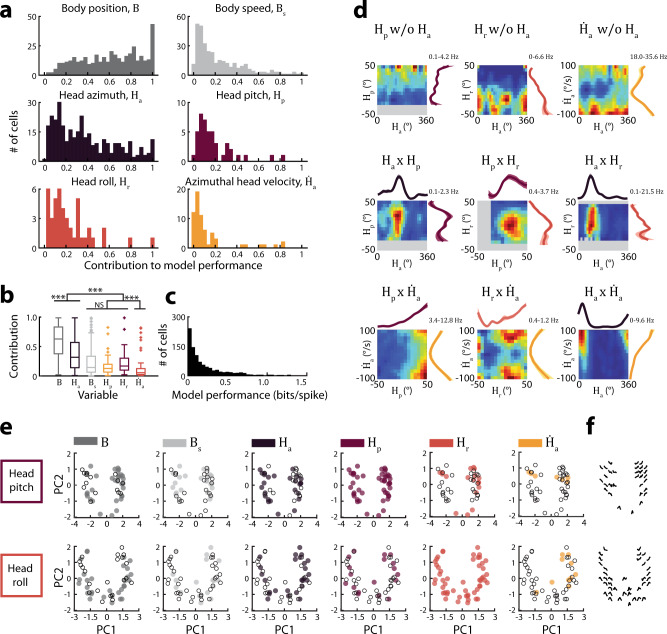
Conjunctive tuning to head position and conventional navigational variables. **a** The contribution of each variable to model performance (log-likelihood increase (LLI) in information compared to a mean firing rate model; median [1st–3rd quartile], *n* = # of cells significantly encoding the variable of interest and at least one other variable; *B* = 0.64 [0.38–0.84], *n* = 394; *H*_*a*_ = 0.32 [0.14–0.58], *n* = 327; *B*_*s*_ = 0.15 [0.06–0.34], *n* = 332; *H*_*p*_ = 0.13 [0.07–0.18], *n* = 44; *H*_*r*_ = 0.17 [0.10–0.31], *n* = 44; $\dot H_a$ = 0.06 [0.02–0.13, *n* = 64]). **b** Comparison of variable contributions. Boxplots; box, interquartile range; solid line, median; whiskers, range; outliers plotted separately _*(*_number of cells as in (**a**); *Χ*^*2*^ = 410.3, *P* = 1.8e−86, df = 5; Kruskal–Wallis test followed by post hoc two-sided Wilcoxon rank-sum comparisons with α = 0.0033 after Bonferroni correction for multiple comparisons). ***for all significant pairwise comparisons, *P* < 0.00055. **c** The spike-normalized average log-likelihood increase in information across 10 folds of held-out data for all cells significantly encoding at least one variable (median [1st–3rd quartile]; 0.11 [0.04–0.26], *n* = 869). **d** Joint tuning curves. Examples where the cell encoded variable *a* but not *b* are labeled “*a* w/o b” (w/o; without). Examples where the cell encoded both variables are labeled *“a* × *b”*. Minimum and maximum firing rates are shown at top, with warmer colors indicating higher firing rates. Unvisited bins are shown in gray. Only model-derived tuning curves for significantly encoded variables are shown. **e** For each head position variable we constructed a two-dimensional “tuning curve profile space” (Methods, Supplementary Fig. 11). Within a plot, each circle represents a cell whose tuning curve was projected onto the first two principal components. Subplots are colored according to whether the cell significantly encoded each additional variable. **f** Example tuning curves from a subset of cells are shown at the cell’s position in the tuning curve profile space. *B*, body position; *B*_*s*_, body speed; *H*_*a*_, azimuthal head direction; *H*_*p*_, pitch; *H*_*r*_, roll; $\dot H_a$, azimuthal head velocity.

We assessed the degree to which neural activity in MEC was explained by head pitch position, roll position, and azimuthal velocity, compared to other well-established signals, such as body position, body speed, and azimuthal head direction, by quantifying the unique explanatory contribution of each individual variable^27^. We first computed the model fit for all cells significantly encoding more than one navigational variable. We then created a series of reduced models in which each selected variable was removed and the model re-fit. Reduced models resulted in a decrement of spike prediction accuracy. Comparing this decrease to the original, full model yielded the explanatory power added by the removed variable while accounting for potential correlations among encoded variables. Similar to previous work, we observed that body position was the strongest predictor of MEC spiking, followed by azimuthal head direction (*Χ*^*2*^ = 410.3, *P* = 1.7e−86, df = 5, Kruskal–Wallis test; pairwise comparisons: *B* versus *H*_*a*_: *P* = 1.3e−26, *H*_*a*_ versus remaining variables: *P* = 3.5e−4; Fig. 3a, b)^27^. Under our experimental conditions, head pitch and roll position each contributed less to MEC spiking than azimuthal head direction (*P* = 3.7e−7), but were on par with body speed, a well-established navigational variable encoded by MEC neurons (*P* = 0.38)^30^. Azimuthal head velocity contributed the least of all the navigational signals (Supplementary Fig. 9).

Given that a majority of cells in MEC encoded multiple variables (Figs. 1c, d, 3d, and Supplementary Fig. 10), we examined whether cells with similar tuning to head position or velocity encoded similar sets of other variables. Such structure could reveal functional organization within the circuit^27^. To quantify tuning similarity, we used PCA to generate a set of two-dimensional “tuning curve profile spaces”, in which similar tuning curve shapes for each navigational variable are located close to one another (Fig. 3e, f and Supplementary Fig. 11; Methods). Each cell’s tuning curve was projected onto this space. For each variable, we then labeled points according to whether or not cells encoded an additional variable of interest. For example, in one iteration of this procedure, all pitch-encoding cells were labeled if they also encoded body position (Fig. 3e, top left). By assessing whether labeled points were significantly clustered, we evaluated whether cells with similar tuning curves to one navigational variable tended to also encode similar additional variables. Across all possible combinations, we did not observe any clustering in the assigned labels (all Bonferroni corrected *P* > 0.005; Methods; Supplementary Fig. 11). Thus, although some neurons exhibited similar tuning to body position, body speed, azimuthal head direction, pitch position, roll position, or azimuthal velocity, they did not otherwise exhibit similar coding features such as the set of variables encoded. This is consistent with prior work that considered a restricted set of variables^27^, and highlights that MEC not only exhibits a high degree of diversity amongst the tuning curves for navigational variables, but also in the way different signals are combined in individual neurons.

### Entorhinal neurons encode eye movements

**Fig. 4:**
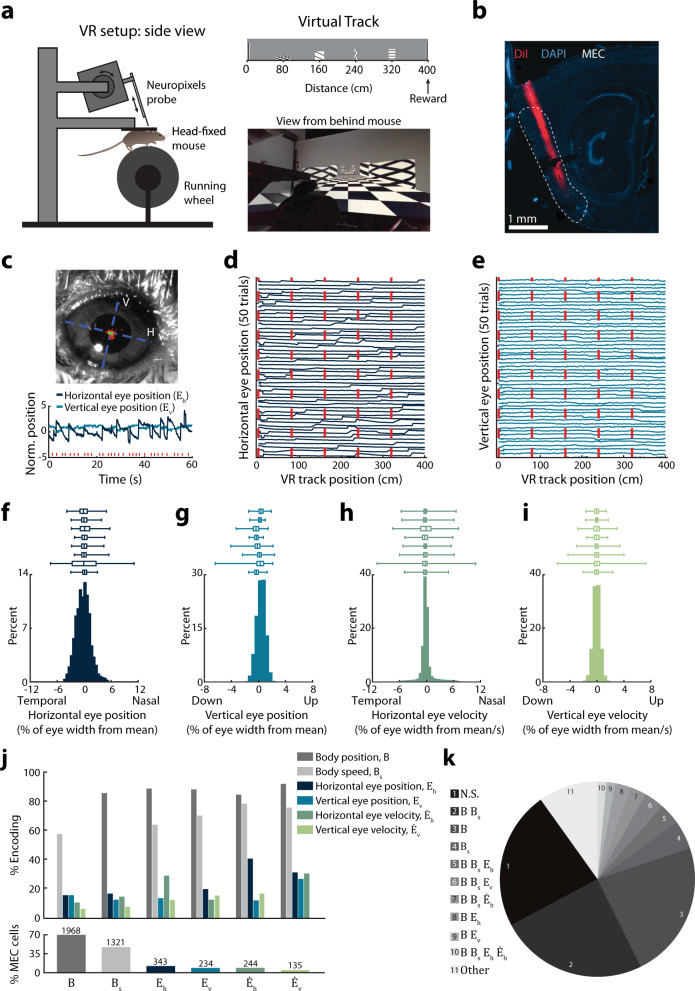
MEC tuning to eye position. **a** VR and Neuropixels probe setup. **b** Histology showing Neuropixels probe placement (red) in MEC. **c** Top: Photograph of eye. Red, pupil positions from one session. Green, positions within the inner 98% of the distribution. H/V, horizontal/vertical eye axes. Bottom: Example eye position traces. Red ticks, landmark occurrences. **d** Horizontal eye position over 50 trials. Red lines, landmarks. **e** As in (**d**) but for vertical eye position. **f** Horizontal eye positions in one session. Boxplots: distributions in individual mice (*n* = 8), averaged across sessions. Box, interquartile range; vertical line, median; whiskers extend from the 1st to 99th percentiles. Mean ± sem across mice; units = % of eye width from mean position: 1st percentile = −3.50 ± 0.61, 25th percentile = −0.97 ± 0.27, median = −0.11 ± 0.032, 75th percentile = 0.83 ± 0.26, 99th percentile = 5.28 ± 0.87. **g** As in (**f**) but for vertical eye position. 1st percentile = −2.74 ± 0.64, 25th percentile = −0.35 ± 0.11, median = −0.035 ± 0.11, 75th percentile = 0.28 ± 0.11, 99th percentile = 1.60 ± 0.21. **h**, as in (**f**) but for horizonal eye velocity. Units = % of eye width from mean position/sec: 1st percentile = −6.19 ± 0.74, 25th percentile = −0.58 ± 0.091, median = −0.11 ± 0.029, 75th percentile = 0.36 ± 0.12, 99th percentile = 6.87 ± 0.66. **i** As in (**f**) but for vertical eye velocity. 1st percentile = −2.83 ± 0.55, 25th percentile = −0.29 ± 0.025, median = 0.013 ± 0.010, 75th percentile = 0.31 ± 0.031, 99th percentile = 3.15 ± 0.68. **j** Top: Proportion of cells jointly encoding variables. Bottom: Percentage of cells significantly encoding each variable with cell count at top. **k** Tuning to multiple variables (*n* = 2861 cells). “Other”, combinations observed in <10 cells. Few cells encoded eye position, eye velocity, or body speed alone (horizontal eye position, 0.3%, vertical eye position, 0.1%, horizontal eye velocity, 0.2%, vertical eye velocity, 0.2%, body speed 5%). 23% of MEC cells encoded body position non-conjunctively. N.S., no significant tuning.

Our results from freely foraging mice revealed that neural activity in MEC varies with head position and velocity about multiple axes. However, for visual information to be accurately interpreted in allocentric coordinates, the brain must also account for the position and movement of the eyes. Thus, we next investigated whether MEC neurons also carry signals related to eye movements. In freely moving animals, eye movements are coordinated with head movements through mechanisms such as the vestibulo-ocular reflex^31^. To isolate the potential contribution of eye movements to MEC activity, we measured eye movements in head-fixed mice navigating in virtual reality while recording MEC activity using Neuropixels probes acutely implanted in either the right or left hemisphere (Fig. 4a, b, Methods*;* Supplementary Fig 1). Eye movements were monitored in the right eye using a video-capture system as mice traversed a 400 cm long virtual reality linear track to receive liquid rewards. Eye position and eye velocity were quantified using a distance metric normalized to the width of each mouse’s eye (e.g. 1 position unit = 1/100 of the eye width). Mice traversed two virtual tracks: one with a checkered floor pattern to provide optic flow plus five distinct, evenly spaced visual cues (landmarks) to provide positional information (Fig. 4a), and another that contained only the checkered floor pattern (Fig. 7a).

On the virtual track with landmarks, head-fixed mice moved their eyes considerably, as reported previously^6,7,32^ (Fig. 4c–e). An LN model containing eye movement-related variables (position and velocity of the eye along its horizontal [*E*_*h*_, $\dot E_h$; nasal-temporal] and vertical [*E*_*v*_, $\dot E_v$; dorsal-ventral] axes, Fig. 4c) along with (virtual) body position and speed (*B* and *B*_*s*_) was used to characterize the activity of 2861 MEC neurons from 8 mice over 11 recording sessions. Although eye position and velocity on both the horizontal and vertical axes were more correlated than chance with body position and running speed in the virtual track, the large variance in eye movements across the track enabled the statistical separation of neural signals related to eye movements (Supplementary Fig. 12). 711/2861 MEC cells [25%] carried signals related to eye movements: 343 cells [12%] encoded horizontal eye position, 234 cells [9%] encoded vertical eye position, 244 cells [9%] cells encoded horizontal eye velocity, and 135 cells [5%] encoded vertical eye velocity. The majority of cells encoding eye movements conjunctively encoded body position or speed (96%), and a subset of cells encoded multiple parameters of eye movements (28%, Fig. 4j, k).

**Fig. 5:**
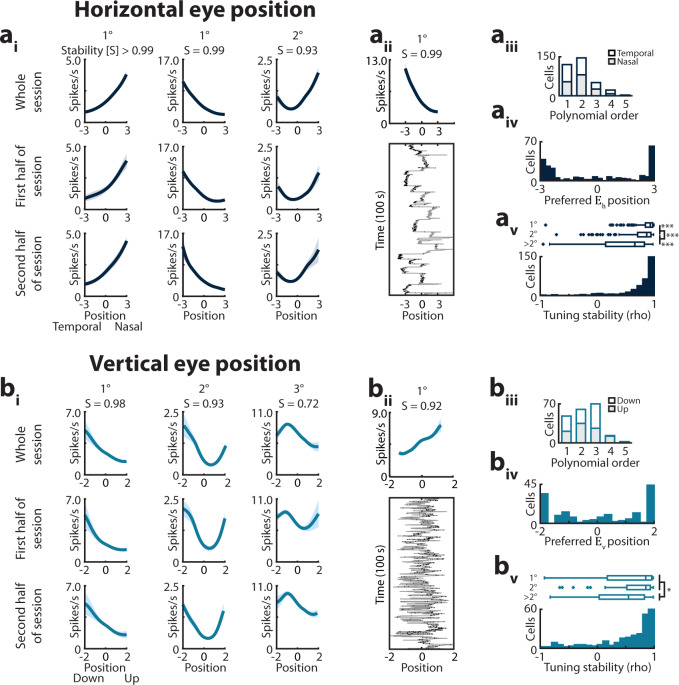
Heterogenous tuning to eye position. **a**_**i**_ Model-derived tuning curves (mean ± sem of 10 model folds) for cells significantly encoding horizontal eye position (*E*_*h*_; Supplementary Figs 5–8). The stability (“S”) and the class of the tuning curve (1° = linear, etc.) are indicated at top. **a**_**ii**_ Bottom_:_ Spiking from an *E*_*h*_-encoding cell. Gray trace, *E*_*h*_. Black dots, spikes. Top: The associated tuning curve. **a**_**iii**_ Summary of tuning curve classes. Gray bars indicate nasal/temporal preference. **a**_**iv**_ Preferred position for cells non-linearly encoding *E*_*h*_. **a**_**v**_ Stability of tuning to *E*_*h*_. Boxplots: linearly tuned cells were significantly more stable than those exhibiting greater curvature (median [1st–3rd quartile]; 1° = 0.93 [0.85–0.98]; 2° = 0.87 [0.71–0.95]; >2° = 0.66 [0.15–0.83]; *Χ*^*2*^ = 69.59, *P* = 7.7e−16, df = 2; 1° versus 2°, *P* = 2.6e−5, 1° versus > 2°, *P* = 1.8e−15, 2° versus > 2°, *P* = 4.1e−8; Kruskal–Wallis test with post hoc two-sided Wilcoxon rank-sum comparisons with α = 0.0167 after Bonferroni correction for multiple comparisons). **b**_**i**_ Tuning curves for cells encoding vertical eye position (*E*_*v*_). **b**_**ii**_ Spiking from an *E*_*v*_-encoding cell. **b**_**iii**_ Summary of tuning curve classes. Bar color indicates upward/downward preference. **b**_**iv**_ Preferred position for cells non-linearly tuned to *E*_*v*_. **b**_**v**_ Stability of tuning to *E*_*v*_. Boxplots: linearly or quadratically tuned cells were significantly more stable than those exhibiting greater curvature (median [1st–3rd quartile]; 1° = 0.86 [0.18–0.97]; 2° = 0.86 [0.53–0_._94]; >2° = 0.56 [0.04_–_0.84]; *Χ*^*2*^ = 17.17, *P* = 1.9e−4, df = 2; 1° versus 2° *P* = 0_._87, 1° versus > 2° *P* = 0.012, 2° versus > 2° *P* = 0_._0001_;_ Kruskal–Wallis test with post hoc two-sided Wilcoxon rank-sum comparisons with α = 0.0167 after Bonferroni correction for multiple comparisons). Boxplots: box, interquartile range; solid line, median; whiskers, range; outliers plotted separately.

Cells encoding horizontal or vertical eye position displayed a diversity of tuning curve profiles (Fig. 5a, b). To characterize this diversity, we applied the same classification framework used to analyze neural tuning to head movements and grouped cells according to the order of the best-fit polynomial (Fig. 5a_iii_, b_iii_ *E*_*h*_ *n* = 343/343, *E*_*v*_ *n* = 177/234 cells). Of the cells encoding horizontal eye position, a similar proportion fired maximally for nasal versus temporal eye positions (nasal-preferring *n* = 170/343, *Z* = 0.11, *P* = 0.91, Fig. 5a_iii_), even when considering each recorded hemisphere independently (ipsilateral hemisphere: nasal-preferring *n* = 38/87, *Z* = 1.07, *P* = 0.28; contralateral hemisphere: nasal-preferring *n* = 132/256, *Z* = 0.43, *P* = 0.66). Overall, the preferred horizontal eye positions of non-linearly tuned cells were broadly distributed (Fig. 5a_iv_). Likewise, of cells encoding vertical eye position, similar proportions fired maximally for upward versus downward eye positions (upward-preferring *n* = 88/177, *Z* = 0, *P* = 1, Fig. 5b_iii_), and the preferred vertical eye positions of non-linearly tuned cells were broadly distributed (Fig. 5b_iv_). Tuning was stable across the recording session (correlation coefficient, median [1st–3rd quartile]; *E*_*h*_ = 0.87 [0.67–0.95], *n* = 343; *E*_*v*_ = 0.72 [0.21–0.91], *n* = 177), with tuning to horizontal eye position significantly more stable than tuning to virtual body position, and tuning to vertical eye position similar to that of virtual body position (0.71 [0.52–0.85], *n* = 1968; *Χ*^*2*^ = 103.51, *P* = 3.3–23, df = 2, Kruskal–Wallis test; pairwise comparisons: *E*_*h*_ versus *B*: *P* = 8.6e−25, *E*_*v*_ versus *B*: *P* = 0.91; Fig. 5a_v_, b_v_).

**Fig. 6:**
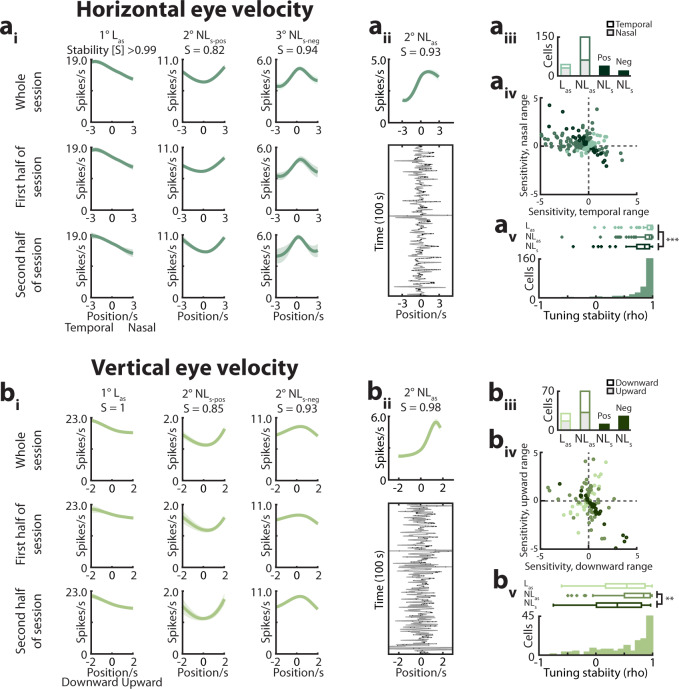
Heterogenous tuning to eye velocity. **a**_**i**_ Model-derived tuning curves (mean ± sem of 10 model folds) for cells significantly encoding horizontal eye velocity ($\dot E_{h}$; Supplementary Figs. 5–8). The stability (“S”) and tuning curve class is indicated at top (L_as_, linear-asymmetric; NL_as_, nonlinear-asymmetric; NL_s_, nonlinear-symmetric; Pos, positive; Neg, negative). **a**_**ii**_ Bottom: Spiking from an $\dot E_{h}$-encoding cell. Gray trace, *E*_*h*_. Black dots, spikes. Top: The associated tuning curve. **a**_**iii**_ Summary tuning curve classes. Bar color indicates nasal/temporal preference. **a**_**iv**_ Cells’ sensitivity in the temporal versus nasal rotational ranges. Units = spikes/s/normalized eye width/s.  **a**_**v**_ Stability of tuning to $\dot E_{h}$. Boxplots: linear-asymmetric and nonlinear-asymmetric cells were significantly more stable than nonlinear-symmetric cells (median [1st–3rd quartile]; L_as_ = 0.96 [0.91–0.99]; NL_as_ = 0.94 [0.87–0.98]; NL_s_ = 0.86 [0.72–0.93]; *Χ*^*2*^ = 19.30, *P* = 6.4e−5, df = 2; L_as_ versus NL_as_, *P* = 0.12, L_as_ versus NL_s_, *P* = 0.00028, NL_as_ versus NL_s_, *P* = 0.00012; Kruskal–Wallis test with two-sided post hoc Wilcoxon rank-sum comparisons with α = 0.0167 after Bonferroni correction for multiple comparisons). **b**_**i**_ Tuning curves for cells significantly encoding vertical eye velocity ($\dot E_v$). **b**_**ii**_ Spiking from an $\dot E_{v}$-encoding cell. **b**_**iii**_ Summary of tuning curve classes. Bar color indicates upward/downward preference. **b**_**iv**_  Cells' sensitivity in the upward versus downward velocity ranges. Units = spikes/s/normalized eye width/s. **b**_**v**_ Stability of tuning to $\dot E_v$. Boxplots: nonlinear-asymmetric cells were significantly more stable than nonlinear symmetric cells (median [1st–3rd quartile]; L_as_ = 0.55 [0.18–0.87]; NL_as_ = 0.85 [0.51–0.96]; NL_s_ = 0.38 [0.02–0.81]; *Χ*^*2*^ = 11.37, *P* = 0.0034, df = 2; L_as_ versus NL_as_, *P* = 0.048, L_as_ versus NL_s_, *P* = 0.33, NL_as_ versus NL_s_, *P* = 0.0014; Kruskal–Wallis test with post hoc two-sided Wilcoxon rank-sum comparisons with α = 0.0167 after Bonferroni correction for multiple comparisons). Boxplots: box, interquartile range; solid line, median; whiskers, range; outliers plotted separately.

Like eye position, eye velocity predicted neural activity in a manner well-characterized by polynomials ($\dot E_h$ *n* = 244/244, $\dot E_v$ *n* = 131/135 cells; Fig. 6a, b, Supplementary Figs. 7, 8). In a small subpopulation of eye velocity-encoding cells, firing varied linearly with eye velocity (linear-asymmetric [L_as_]; $\dot E_h$ *n* = 43/244, $\dot E_v$ *n* = 29/131 cells). Linear-asymmetric cells encoding horizontal eye velocity showed a preference for nasal-directed movements (nasal-preferring *n* = 30/43, *Z* = 2.44, *P* = 0.014), with similar preferences observed in each hemisphere (ipsilateral hemisphere nasal-preferring *n* = 4/6, contralateral hemisphere *n* = 26/37). Cells encoding vertical eye velocity did not show a preference for upward versus downward movements (upward-preferring *n* = 16/29, *Z* = 0.37, *P* = 0.71; Fig. 6a_iii,iv_, b_iii,iv_). The majority of eye velocity-encoding cells exhibited nonlinear tuning ($\dot E_h$ *n* = 201/244, $\dot E_v$ *n* = 102/131 cells), and were subclassified as nonlinear-asymmetric ([NL_as_]; $\dot E_h$ *n* = 147/244 cells, $\dot E_v$ *n* = 68/131 cells) and nonlinear-symmetric ([NL_s_]; $\dot E_h$ *n* = 54/244 cells, $\dot E_v$ *n* = 34/131 cells) (Fig. 6a_iii,iv_, b_iii,iv_, Supplementary Fig. 8). Nonlinear-asymmetric cells encoding horizontal movement preferred temporal-directed movements (NL_as_−$\dot E_h$ temporal-preferring *n* = 87/147, *Z* = 2.22, *P* = 0.026), with similar proportions of cells preferring temporal-directed movements in both the ipsilateral and contralteral hemispheres (ipsilateral hemisphere temporal-preferring *n* = 19/33, contralateral hemisphere = 68/114). Cells encoding vertical moment did not exhibit a directional preference (NL_as_−$\dot E_v$ downward-preferring *n* = 31/68, *Z* = −0.61, *P* = 0.54). Tuning to horizontal eye velocity was significantly more stable than that to virtual body speed, while tuning to vertical eye velocity was significantly less stable (median [1st–3rd quartile]; $\dot E_h$ = 0.93 [0.82–0.98], *n* = 244; $\dot E_v$ = 0.74 [0.21–0.92], *n* = 131; *B*_*s*_ = 0.84 [0.60–0.94], *n* = 1321; *Χ*^*2*^ = 90.78, *P* = 1.9–20, df = 2, Kruskal–Wallis test; pairwise comparisons: $\dot E_h$ versus *B*_*s*_: *P* = 6.5e−18, $\dot E_v$ versus *B*_*s*_: *P* = 0.00061; Fig. 6a_v_, b_v_).

**Fig. 7:**
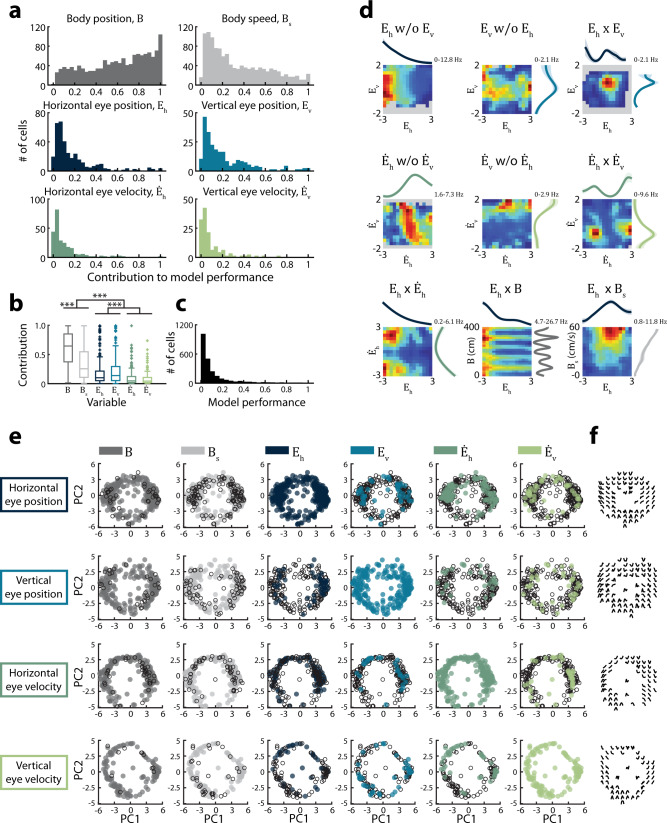
Conjunctive tuning to eye movement-related and conventional navigational variables. **a** The contribution of each variable to model performance (log-likelihood increase (LLI) in information compared to a mean firing rate model; *n* = # of cells significantly encoding the variable of interest and at least one other variable; median [1st–3rd quartile], *B* = 0.65 [0.38–0.86], *n* = 1319; *B*_*s*_ = 0.26 [.11–0.55], *n* = 1187; *E*_*h*_ = 0.10 [0.05–0.22], *n* = 335; *E*_*v*_ = 0.14 [0.05*–*0.30], *n* = 231; $\dot E_h$ = 0.05 [0.02–0.12], *n* = 237; $\dot E_v$ = 0.04 [0.02–0.11], *n* = 132). **b** Comparison of variable contributions (# of cells as in (**a**); *Χ*^*2*^ = 1.3e3, *P* = 5.5e−276, df = 5; Kruskal–Wallis test followed by post hoc Wilcoxon two-sided rank-sum comparisons with α = 0.0033 after Bonferroni correction for multiple comparisons). ***for all significant pairwise comparisons, *P* < 1.9e−10. Boxplots; box, interquartile range; solid line, median; whiskers, range; outliers plotted separately. **c** The spike-normalized average log-likelihood increase in information across 10 folds of held-out data for all cells significantly encoding at least one variable (median [1st–3rd quartile]; 0.05 [0.02–0.11], *n* = 2191). **d** Joint tuning curves. Labels as in Fig. 3d. Minimum and maximum firing rates shown at top, with warmer colors indicating higher firing rates. Unvisited bins are shown in gray. Only model-derived tuning curves for significantly encoded variables are shown. **e** For each eye movement-related variable we constructed a two-dimensional “tuning curve profile space”. Within a plot, each circle represents a cell whose tuning curves were projected onto the first two principal components (Methods, Supplementary Fig. 11). Subplots are colored according to whether the cell significantly encoded each additional variable. **f** Example tuning curves from a subset of cells are shown at the cell’s position in the tuning curve profile space. *B*, body position; *B*_*s*_, body speed; *E*_*h*_, horizontal eye position; *E*_*v*_, vertical eye position; $\dot E_h$, horizontal eye velocity; $\dot E_v$, vertical eye velocity.

Quantification of the contributions of each behavioral variable revealed that body position and speed influenced MEC spiking more strongly than eye movement-related signals (*Χ*^*2*^ = 1.3e3, *P* = 5.5e–276, df = 5; Kruskal–Wallis test; Fig. 7a–c; Supplementary Fig. 9). The contributions of horizontal and vertical eye position were greater than those of horizontal and vertical eye velocity (*P* = 1.9e–10). A population of cells conjunctively encoded eye position or velocity along with the traditional navigational variables, body position, and speed (Fig. 7d; Supplementary Fig. 10). As with cells encoding head movements, cells that exhibited similar tuning to eye position or velocity did not tend to encode similar additional variables (Fig. 7e, f; Supplementary Fig. 11). These data suggest that both eye position and velocity are integrated into the heterogeneous coding structure of MEC^19,27^.

### Encoding of eye position and eye velocity persists in the absence of visual landmark features

**Fig. 8:**
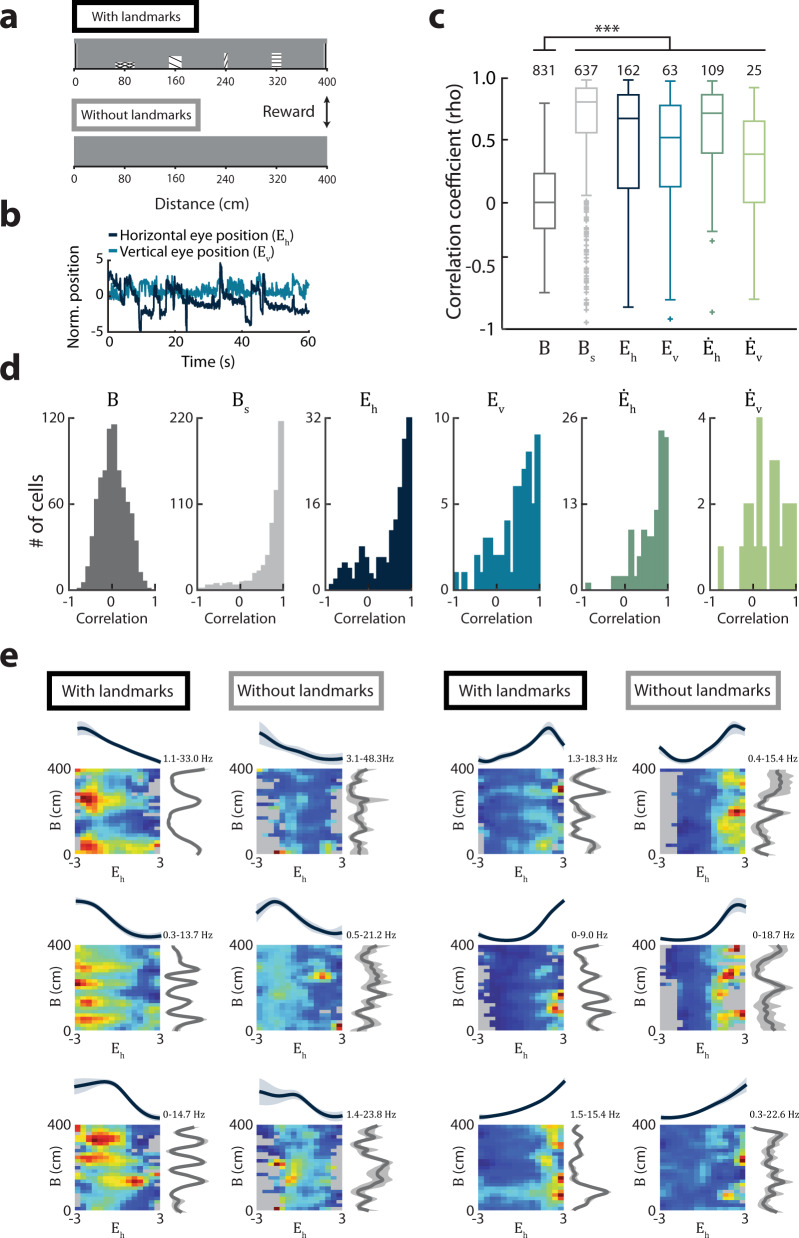
Tuning to eye position and velocity does not depend on visual information. **a** On interleaved blocks of trials, mice (*n* = 6) ran either on a track with five distinct visual landmarks (“With landmarks”), or a track of the same length but devoid of visual landmarks (“Without landmarks”). In both tracks, the floor was checkerboard-patterned. **b** Example traces of *E*_*h*_ and *E*_*v*_ position as an animal traversed the track without visual landmarks. **c** Boxplots showing the correlation coefficients between cells’ tuning curves in both tracks. Box, interquartile range; solid line, median; whiskers, range; outliers plotted separately. Correlations were computed for cells significantly encoding each variable on the landmark track (*n* for each variable is shown at top of plots). Position tuning curves were nearly uncorrelated between the tracks (median [1st–3rd quartile], *B* = 0.02 [−0.19–0.25], whereas the tuning curves for cells encoding all eye movement-related variables and body speed were preserved to a significantly greater extent (median [1st–3rd quartile*]*, *B*_*s*_ = 0.82 [0.58*–*0.93], *E*_*h*_ = 0.69 [0.13–0.88], *E*_*v*_ = 0.54 [0.14–0.79], $\dot E_h$ = 0.73 *[*0^.^41–0.88], $\dot E_v$ = 0.40 [0.02–0.67]; *Χ*^*2*^ = 748.27, *P* = 1.8e-159, df = 5, *B* versus each other variable, all *P* < 0.00024, Kruskal–Wallis test followed by post hoc two-sided Wilcoxon rank-sum comparisons with α = 0.0033 after Bonferroni correction for multiple comparisons. ***P < 0.00024. **d** Histograms indicating the correlation coefficient between tuning curves in both tracks (same data as in (**c**)). **e** Examples of joint tuning curves for cells significantly encoding both horizonal eye position (*E*_*h*_) and body position (*B*) in the landmark condition. The model-derived tuning curves (mean ± sem from 10 model folds) for horizontal eye position and body position are shown at top and right. The minimum and maximum firing rates are shown at top. Unvisited bins are shown in gray. *B*, body position; *B*_*s*_, body speed; *E*_*h*_, horizontal eye position; *E*_*v*_, vertical eye position; $\dot E_h$, horizontal eye velocity; $\dot E_v$, vertical eye velocity.

A large proportion of MEC neurons encoding eye position also encoded body position on the virtual track (Fig. 7d, Supplementary Fig. 10), raising the question of whether MEC neurons are driven by eye movements per se, or whether they might instead be driven by the visual landmark features that occur at specific positions on the track. To distinguish between these possibilities, we analyzed a second dataset in which mice traversed both the virtual track with landmarks (as above) and a second virtual track without landmarks (Fig. 8a, b). Comparison of a given cell’s tuning curve in the presence and absence of landmarks revealed that tuning to body position along the track tended to degenerate or remap in the absence of landmarks (*n* = 1621 cells recorded from 6 sessions in 6 mice; Fig. 8c–e). However, along with body speed, tuning to eye position and eye velocity was preserved across conditions (Fig. 8). This effect was observed even in cells that jointly encoded body position and eye position (Fig. 8e). Combined, this indicates that entorhinal coding for eye movement is robust to changing visual features and may reflect self-motion cues from the eye.

## Discussion

Rodents move their bodies, heads, and eyes substantially during navigation and thus require a repertoire of self-motion signals to compute an accurate allocentric representation of space from sensory inputs. Our experiments revealed neural activity in MEC correlated with the position and movement of the head and eyes about multiple axes, in addition to the previously reported body position, body speed, and azimuthal head direction signals. These novel self-motion signals could allow MEC to locally compute information about the location of visual landmarks and the animal’s position relative to those locations. The heterogeneous and conjunctive representation of these signals is similar to previous observations for body position, body speed, and azimuthal head direction signals in MEC^27^.

There are several possible sources of the head- and eye-movement-related signals in MEC. The head movement-related neural activity could arise from the vestibular system, efference copy of motor commands to the neck, neck proprioceptive signals, or eye movements, all of which correlate with head movements^6,7,31,33^. Signals encoding head and eye movements have been identified in a number of other cortical and subcortical structures^26,28,29,31,34,35^. MEC receives direct input from two areas that carry signals correlated with 3D head position and velocity, the retrosplenial cortex and postsubiculum^36^. The eye movement signals in MEC could originate from the oculomotor circuitry in the brainstem, midbrain, cerebellum, or cortex^37–39^. In mice, recent work identifying cortical control of eye movement by the secondary motor cortex (MOs) makes it a candidate source of the eye-movement-related signals in MEC, since eye-movement signals in MOs are relayed to secondary visual areas including AL^40^, which projects to MEC.

The observation that eye movements are abundant in foraging rodents^6–8^, coupled with our finding that such signals are encoded by MEC neurons, highlights the need to consider eye movements when characterizing the response properties of parahippocampal circuits. In freely moving animals, there is intricate coordination of body, head, and eye movements through mechanisms such as the vestibulo-ocular reflex, vestibulo-collic reflex, and optokinetic reflex^6,7,31^. Moreover, these movements are both driven by and influence sensory inputs, and may also reflect a variety of cognitive variables and behavioral states. These correlations among multiple variables create a challenge in distinguishing the contribution of each to the conjunctive coding of neurons in the medial temporal lobe. For example, the potential confounds associated with head or body direction have been carefully weighed in studies of “splitter” cells, which fire in a place-specific manner dependent on an animal’s future trajectory, or “theta sequences,” which depict alternative routes sweeping forward from an animal’s current position^41,42^. However, it is possible that conjunctive coding of place and eye movement could contribute to such phenomena if eye movements near choice points reflect upcoming behavior. Future experiments that combine statistical modeling approaches with simultaneous tracking of the head, eyes and body in 3D, plus behavioral manipulations to break the correlations among variables, will be required to more fully untangle the signals carried by neurons in the navigation circuitry.

The reference frames for the head- and eye-movement-related signals in MEC could be egocentric or allocentric. The use of a 2D navigation environment in the present experiments leaves unresolved whether MEC tuning to pitch and roll is egocentric (dependent on the angle of the head relative to the neck) or allocentric (dependent on the angle of the head relative to gravity). Likewise, the reference frame of the eye movement-related signals we observed could be egocentric (related to the position of the eye within the orbit) or allocentric (related to the position of the eye in the world, or by the position of the eye relative to a specific visual feature) since the eye-in-head and eye-in-world reference frames are coupled in head-fixed experiments. It is also possible that coding was anchored to the edge of the monitor itself. In primate MEC, gaze cells have been identified that are locked to these different reference frames, with some cells firing for the position of the eyes in the world (gaze direction), and others for the position of the eyes relative to the boundaries of an image^16^. Future work that de-couples these reference frames, either through rotation of a head-fixed animal or through free movement, will be needed to pinpoint the specific role of entorhinal eye signals in navigation-based computations.

Our findings reveal new potential homologies in the navigational coding schemes used between species. First, the observed coding of 3D head movement in mice is consistent with that in the presubiculum of bats^14^. However, in rodent MEC, we observed a greater degree of conjunctive coding, including cells that simultaneously encode position and velocity of the head about multiple axes alongside body position and body speed. Second, the coding of eye position in rodents bears similarities to the coding of eye (or gaze) position observed in the MEC of head-fixed non-human primates. Yet primate gaze signals have been interpreted as playing a role in exploration of visual space analogous to the role of body position signals in rodent exploration of physical space^18^. Hence, the observation that rodent MEC simultaneously represents both body position and eye position raises the possibility that primate and rodent eye-movement signals serve distinct functions.

The joint coding of body position with eye or head position (Figs. 3d, 6d) is reminiscent of a coding scheme observed in primate posterior parietal cortex, wherein eye or head position modulates the amplitude of a cell’s response within its visual receptive field (i.e. “gain fields”)^43,44^. Since their discovery in primate parietal cortex, eye position gain fields have been reported widely throughout the brain, particularly in regions processing visuomotor information, including frontal cortex^44,45^, visual cortex^46,47^, and premotor cortex^48^. Gain modulation has been theorized to support transformations between relevant reference frames^49,50^. For example, posterior parietal eye position gain fields may support the transformation of visual representations from retinotopic-centered coordinates to body-centered coordinates downstream^49,50^. Whereas spatial representations in MEC are allocentric^19–22^, modulation of these signals by head or eye position could facilitate a similar downstream transformation into other reference frames useful for navigation. For example, eye or head modulation of border cells in MEC could contribute to egocentric boundary coding in the dorsomedial striatum^51^. Understanding the functional role of eye and head position signals, both within MEC and in downstream regions, will be an important area of future work.

The presence of eye and head-related signals in MEC highlights the critical importance of self-motion signals, which play multiple functional roles to support entorhinal coding. Self-motion information is necessary to compute the location of environmental landmarks, which anchor the firing patterns of MEC grid cells and border cells^4,22^. Further, self-motion signals can provide an estimate of an animal’s velocity through space, a signal hypothesized by computational works to generate grid cell spatial periodicity^52^. Our findings of a wide repertoire of self-motion signals in MEC raise the possibility that other, yet-to-be-discovered self-motion cues are processed by medial temporal lobe.

## Methods

### Freely moving dataset

#### Animals

Neural recordings in the MEC of freely moving mice were obtained from two cohorts of mice. The first cohort (Cohort 1; *n* = 4 female, 2 male C57BL/6 mice, 179 cells) was equipped with 2 LEDs affixed to the headstage to monitor body position (*B*), body speed (*B*_*s*_), azimuth head direction (*H*_*a*_.), and angular head velocity about the azimuth axis ($\dot H_a$), and an inertial measurement unit (IMU) to monitor head pitch (*H*_*p*_), roll (*H*_*r*_), angular head velocity about the pitch axis ($\dot H_p$), or angular head velocity about the roll axis ($\dot H_r$). The second cohort (Cohort 2; *n* = 20 mice, 1021 cells) was equipped with LEDs only to measure azimuth head movements. This cohort included unpublished data (*n* = 5 male 50:50 hybrid C57BL/6J:129SVEV mice, 228 cells) and 15 mice included in previously published studies (*n* = 2 female C57BL/6J mice, 122 cells from^53^; 7 male 50:50 hybrid C57BL/6J:129SVEVmice, 389 cells from^54^, and 6 male C57BL/6 mice, 282 MEC cells from ref. ^55^).

At the time of surgery, mice ranged between 2 and 12 months in age. After tetrode implantation, mice were house individually in transparent cages on either a reverse light cycle, with testing taking place during the dark phase^54^ or a normal cycle, with testing taking place during the light phase (Campbell et al.^53^, Munn et al.^55^ and unpublished data). Animals were housed in facilities with temperature maintained at 71 degrees Fahrenheit and 40% humidity. For unpublished data in Cohorts 1 and 2, Campbell et al.^53^, and Munn et al.^55^, all procedures were approved by Stanford University’s Administrative Panel on Laboratory animal Care. For data from Eggink et al.^54^ (in Cohort 2), all experiments were performed in accordance with the Norwegian Animal Welfare Act and the European Convention for the Protection of Vertebrate Animals used for Experimental and Other Scientific Purposes and approved by the National Animal Research Authority of Norway.

#### Surgery

For Cohort 1, a Microdrive holding two moveable tetrodes was implanted above the right MEC and a metal bar affixed horizontally across the top of the skull for immobilizing the head during calibration sessions for the accelerometer. For Cohort 2, a Microdrive holding two moveable tetrodes was implanted above the right (*n* = 7) or left MEC (*n* = 13). Anesthesia was induced by an injection of buprenphine (0.1 mg/kg) and maintained with isoflurane (0.5 –3%). Mice were unilaterally implanted with a 2 tetrode Microdrive connected to 17 µm polyimide-coated platinum–iridium (90–10%) wire tetrodes (plated to impedances of 150–250 kΩ at 1 kHz). Tetrodes were implanted at 0.45 mm anterior-posterior from the transverse sinus, 3.28 mm medial-lateral, and 0.8 mm below the dura, angled 0–4 degrees in the posterior direction in the sagittal plane. Small screws, Metabond, and dental cement were used to affix the head bar and Microdive to the animal’s skull. Beginning 5 days after implantation, mice were habituated to the training arena.

#### Data collection

Data were collected during daily sessions lasting 20–60 min, while mice foraged for scattered food (crumbled chocolate cereal) in arenas of varying sizes (box width and length; 100 cm × 100 cm, 50 cm × 50 cm, 70 cm × 70 cm, or 70 cm × 35 cm). The animal was habituated to the testing arena ≥ two weeks before data collection began. For each session in Cohort 1, sessions were preceded by a calibration step, in which the animal was briefly head-fixed (for several minutes) to enforce a pitch and roll angle of 0. The accelerometer values under this condition were recorded and later used to compute pitch and roll (see Section 1.6: Measurement of behavioral variables). For all sessions, black curtains surrounded the black recording boxes, with a white cue located in a constant location midway between the corners of one wall. The test box was cleaned with soapy water followed by odor remover (Nature’s Miracle) between each session.

Mice were connected to the recording equipment (Axona Ltd., St Albans, U.K., version 1.3.0.19) via AC-coupled unity-gain operational amplifiers attached to a counterbalanced cable that allowed free movement through the environment. Recorded signals were amplified 8000 to 25,000 times and bandpass filtered between 0.8 and 6.7 kHz. Triggered spikes were stored to a disk at 48 kHz (50 samples per waveform, 8 bits/sample) with a 32 bit time stamp (clock rate at 96 kHz). To track the mouse’s body and azimuth head position, two light-emitting diodes, one small and one large, were attached to the head stage perpendicular to the rostral-caudal axis of the head and detected by an overhead camera at a sampling rate of 50 Hz. For mice in Cohort 1, an inertial measurement unit (SparkFun 9DoF IMU) was attached to the headstage with one axis approximately perpendicular to the floor plane, and one axis perpendicular to the mouse rostral-caudal axis. Tetrodes were moved ventrally by 25 µm following each recording session.

#### Histology

After the final recording session, electrodes were not moved. In a subset of mice, small electrolytic lesions were made to mark the end of the tetrode track by passing 20 μA current for 16 s on two channels of each tetrode. Mice were then killed with an overdose of pentobarbital and transcardially perfused with 1X phosphate-buffered saline (PBS) followed by 4% paraformaldehyde. Brains were extracted and stored overnight in 4% paraformaldehyde before being transferred into 30% sucrose solution. For slicing, brains were rapidly frozen, cut into 40 µm sagittal sections with a cryostat, mounted and stained. To determine tetrode locations, slices were stained for Cresyl Violet, with the positions of the tips of the recording electrodes determined from digital pictures of the brain sections. In the case of electrolytic lesions, the tetrode location was marked as the center of the lesion. The border between MEC and other cortical regions was determined from post hoc Nissl stained sagittal brain sections and based on the reference Allen Brain Atlas^56^ and The Mouse Brain in Stereotaxic Coordinates^57^. The laminar location of recordings in MEC determined based on cytoarchitecture criteria^57^. Tetrodes were located within layers II/III of MEC.

#### Spike sorting and identification of single units

Spike sorting was performed offline using graphical cluster-cutting software (TINT, version 2.4.6, Axona Ltd., St Albans U.K., www.axona.com). To identify recorded cells, clustering was performed manually in two-dimensional projections of the multidimensional parameter space, using autocorrelation and cross-correlation tools as additional separation tools. Only sessions where the animal covered ≥70% of the environment were used in analyses.

#### Measurement of behavioral variables

Body position within the arena (*B*) was calculated by averaging the *x* and *y* position of the two head-stage mounted LEDs. Body speed (*B*_*s*_) was calculated as the Euclidean distance between sequential body positions over time. Azimuth head direction (*H*_*a*_) was calculated from the arctangent of the two LEDS, and returned the angle of the head around the azimuth axis. Angular azimuthal head velocity ($\dot H_a$) was calculated as the difference in azimuth head direction angles over time.

Pitch and roll angles (*H*_*p*_ and *H*_*r*_), and the angular velocity around the pitch and roll axes ($\dot H_p$ and $\dot H_r$), were computed from the accelerometer signal from the *x*, *y*, and *z* axes of IMU $$\left( {{\mathbf{a}} = \left[ {\begin{array}{*{20}{c}} {a_x} \\ {a_y} \\ {a_z} \end{array}} \right]} \right)$$. Raw values of **a** are by default given in a specific coordinate system *C*_1_, where pitch and roll are measured as deflections from the orientation of the IMU when lying flat on a table. As the IMU was not in this orientation when fixed to the mouse’s head, we transformed the raw signal into a second coordinate system *C*_2_ in which pitch and roll were measured as deflections from the IMU orientation when the mouse was head-fixed with its interaural and rostral-caudal axes perpendicular to one another and parallel to the floor. To do this, we computed a matrix *R*_1→2_ that transformed the raw data ${\mathbf{a}}_{C_1}$ into the appropriately transformed data ${\mathbf{a}}_{C_2}$: ${\mathbf{a}}_{C_2} = R_{1 \to 2}{\mathbf{a}}_{C_1}$. The matrix *R*_1→2_ was defined as the inverse of the product of a roll-rotation and a pitch-rotation matrix:

1 $$\begin{array}{*{20}{c}} {R_{1 \to 2} = \left( {R_{{\rm{roll}}}\left( {\theta _r} \right)R_{{\rm{pitch}}}\left( {\theta _p} \right)} \right)^{\rm{T}}} \end{array}$$

where

2 $$\begin{array}{*{20}{c}} {R_{{\rm{roll}}}\left( {\theta _r} \right) = \left[ {\begin{array}{*{20}{c}} 1 & 0 & 0 \\ 0 & {\cos \left( {\theta _r} \right)} & {\sin \left( {\theta _r} \right)} \\ 0 & { - \sin \left( {\theta _r} \right)} & {\cos \left( {\theta _r} \right)} \end{array}} \right]} \end{array}$$

and

3 $$\begin{array}{*{20}{c}} {R_{{\rm{pitch}}}\left( {\theta _p} \right) = \left[ {\begin{array}{*{20}{c}} {\cos \left( {\theta _p} \right)} & 0 & { - {\mathrm{sin}}(\theta _p)} \\ 0 & 1 & 0 \\ {\sin \left( {\theta _p} \right)} & 0 & {\cos \left( {\theta _p} \right)} \end{array}} \right]} \end{array}$$

Both matrices, when applied to a vector **a**, rotate it along the prescribed axes by *θ* radians.

We computed the roll and pitch angles (*θ*_*r*_ and *θ*_*p*_ respectively) following:

4 $$\begin{array}{*{20}{c}} {\theta _r = \arctan \left( {\frac{{a_{y,C_1}}}{{a_{z,C_1}}}} \right)} \end{array}$$

and

5 $$\begin{array}{*{20}{c}} {\theta _p = \arctan \left( {\frac{{ - a_{x,C_1}}}{{\sqrt {(a_{y,C_1}^2 + a_{z,C_1}^2)} }}} \right)} \end{array}$$

To compute ${\mathbf{a}}_{C_1}$, we took the average over the accelerometer values recorded while the animal was in the head-fixed calibration stage implemented prior to collecting spike and behavioral data. This calibration was performed prior to every recording session. Once we computed ${\mathbf{a}}_{C_2}$, we then computed pitch and roll values following the procedure outlined in^7^. First, we defined normal vectors to the pitch and roll planes: $${\mathbf{n}}_{{\mathbf{r}}{\mathrm{oll}}} = \left[ {\begin{array}{*{20}{c}} 0 \\ 1 \\ 0 \end{array}} \right]$$, $${\mathbf{n}}_{{\mathrm{pitch}}} = \left[ {\begin{array}{*{20}{c}} 1 \\ 0 \\ 0 \end{array}} \right]$$, as well as a “starting” vector from which pitch and roll angles were measured (corresponding to the head-fixed head-orientation), $${\mathbf{s}} = \left[ {\begin{array}{*{20}{c}} 0 \\ 0 \\ 1 \end{array}} \right]$$ (order of the entries and their correspondence to pitch/roll are defined by the specific IMU). We projected ${\mathbf{a}}_{C_2}$ onto the plane defined by pitch or roll by computing:

6 $$\begin{array}{*{20}{c}} {proj_n\left( {{\mathbf{a}}_{C_2}} \right) = {\mathbf{a}}_{C_2} - \frac{{n^T{\mathbf{a}}_{C_2}}}{{norm\left( n \right)}}n} \end{array}$$

Next, we computed the final pitch or roll angle, *θ*_*r*,final_ (*H*_*p*_) or *θ*_*p*,final_ (*H*_*r*_) as the angle between the starting vector, and the projected vector:

7 $$\begin{array}{*{20}{c}} {\theta _{{\rm{final}}} = \arccos \left( {\frac{{{\mathbf{s}}^{\rm{T}}{\rm{proj}}_n\left( {{\mathbf{a}}_{C_2}} \right)}}{{{\rm{norm}}\left( {\mathbf{s}} \right). \ast {\rm{norm}}\left( {{\rm{proj}}_n\left( {{\mathbf{a}}_{C_2}} \right)} \right)}}} \right)} \end{array}$$

*H*_*p*_ and *H*_*r*_ values were time-synchronized to behavioral data gathered from the LEDs and to the neural spike data (both collected via the Axona digital data acquisition system, http://209.61.196.245/products/digital) from TTL pulses sent from the Arduino that collected the IMU data. Pulses were received by Axona through an I/O pinout, and were sent whenever IMU data were acquired. The pulses were recorded in both the Axona datastream and the IMU datastream (saved via a Processing script that read directly from the serial monitor); comparison of these pulses then allowed us to properly synchronize the position, IMU, and neural spike data. *H*_*p*_ and *H*_*r*_ values were sampled at ~30 Hz and then linearly interpolated to match the sampling time of behavioral data (50 Hz). Spike times were recorded to high precision (~1 KHz), and were binned to achieve a vector of spike counts per 20 ms bin per cell.

To verify that the IMU was not drifting appreciably over the course of the recording session, we recorded values while the IMU was stationary on a table for 70 min. We found that values drifted < 1 degree (0.3 degrees for pitch, and 0.4 degrees for roll), and the standard deviation of values over the recording session was < 1 degree for both pitch and roll.

Figure 1b shows the distributions of pitch, roll, and azimuthal angular velocity explored by each animal. We observed the following distributions for pitch angular velocity and roll angular velocity: Mean ± sem across mice; pitch velocity: 2.5th percentile = −303.8 ± 19.9°/s, 25th percentile = −49.6 ± 7.2°/s, median = 0.1 ± 0.06°/s, 75th percentile = 50.3 ± 7.2°/s, 97.5th percentile = 300.8 ± 20.6°/s (*n* = 6 mice); roll velocity: 2.5th percentile = −284.0 ± 20.8°/s, 25th percentile = −45.2 ± 6.8°/s, median = 0.04 ± 0.03°/s, 75th percentile = 45.1 ± 7.0°/s, 97.5th percentile = 284.4 ± 21.2°/s (*n* = 6 mice).

### Head-fixed dataset

#### Animals

Eight female C57BL/6 mice, age 2–6 months at the time of surgery, were used for head-fixed Neuropixels probes recordings. After surgery, mice were house individually in transparent cages on a normal light cycle, with testing taking placing during the light phase. Animals were housed in facilities with temperature maintained at 71 degrees Farenheit and 40% humidity. Mice had ad libitum access to food. Prior to behavioral training mice were put onto water deprivation, where they received 0.8 mL of water each day; their weights were monitored daily to ensure that they remained above 85% of baseline. All procedures were approved by Stanford University’s Administrative Panel on Laboratory animal Care.

#### VR setup

In the VR setup, head-fixed mice ran on a 6-inch diameter foam roller (ethylene vinyl acetate) constrained to rotate around one axis. Rotation was measured by a high-resolution quadrature encoder (Yumo, 1024 P/R) and processed by a microcontroller (Arduino UNO). The virtual environment was generated using commercial software (Unity 3D) and updated according to the motion signal. The virtual reality software output a synchronizing pulse every frame via an Arduino UNO, which was recorded alongside electrophysiological data in SpikeGLX (version 20190214). The virtual scene was displayed on three 24” monitors surrounding the mouse. The gain of the linear transformation from ball rotation to translation along the virtual track was calibrated so that the virtual track was 4 m long. The virtual track was an infinitely repeating sequence of 5 landmarks (towers). At the reward tower, the mouse received a water reward (~2 µl). Water rewards were delivered using a solenoid (Cole Parmer) triggered from the virtual reality software, generating an audible click with water delivery.

#### Headbar surgery and craniotomies

Anesthesia was induced with isoflurane (4%; maintained at 1.75%) followed by injection of buprenorphrine (0.1 mg/kg). Fiducial marks were made on the skull ± 3.3 mm lateral from the midline and approximately 4 mm posterior from Bregma. A ground screw was affixed to the skull approximately 2.5 mm anterior and 1.5 mm lateral from Bregma on the left side. A stainless steel headbar was attached to the skull using Metabond and the rest of the exposed skull was covered with Metabond. Transparent Metabond was used to allow visualization of the fiducial marks, which would later guide craniotomies and probe placement. After training, were again anesthetized with isoflurane and the Metabond and skull were shaved down with a dental drill, posterior to the fiducial mark on both sides. Bilateral craniotomies, roughly 500 *μ*m in diameter, were made posterior to the fiducial mark, exposing the transverse sinus. Plastic rings cut from pipette tips (~4 mm diameter) were affixed to the skull around each craniotomy using Metabond. These rings served as wells to hold saline and a layer of silicone oil during recording, which prevented the craniotomy from drying out. Craniotomies were covered with KwikCast and the mouse recovered overnight before the first recording session. A maximum of three recording sessions were performed per hemisphere, each on a different day, for a maximum of six recording sessions on six days total.

#### VR training

After headbar implantation, mice recovered for three days and were given Baytril (10 mg/kg) and Rimadyl (5 mg/kg) daily. After three days, they were taken off Baytril and Rimadyl and put on water deprivation. They received 0.8 mL of water each day and their weights were monitored to ensure that they remained above 85% of baseline. Mice were then habituated to the running wheel in the VR setup and trained to run down a virtual linear hallway for water rewards over the course of 2 weeks.

#### Data collection

Extracellular recordings were made by acutely inserting silicon probes into the craniotomy above the left (*n* = 4 recordings from 3 mice) or right MEC (*n* = 7 recordings from 7 mice) while the animal was head-fixed in the VR setup. One insertion was performed per day per mouse, after which the craniotomy was covered with Kwik Cast and returned to on subsequent days for additional recordings. We performed 3 insertions on each side for a maximum of 6 recordings per mouse over 6 days. We did not observe a difference in the proportions of MEC neurons encoding eye movement-related variables across the left and right hemispheres (proportion of neurons tuned to one or more eye movement-related variables, mean ± sem: left hemisphere = 0.23 ± 0.083, *n* = 4 recordings, right hemisphere = 0.43 ± 0.083, *n* = 7 recordings, *P* = 0.23, Wilcoxon rank-sum test).

#### Neuropixels probes

Recordings were made using single Neuropixels probes^58,59^. 11 recordings from 8 mice, totaling 2861 single units, were made with Neuropixels 1.0 probes (384 recording channels, www.neuropixels.org). Raw voltage traces were filtered, amplified, multiplexed, and digitized on-probe, and recorded using SpikeGLX (billkarsh.github.io*/SpikeGLX*). Voltage traces were filtered between 300 Hz and 10 kHz and sampled at 30 kHz with gain = 500 (AP band) or filtered between 0.5 Hz and 1 kHz and sampled at 2.5 kHz with gain = 250 (LFP band). All recordings were made using the contiguous set of recording channels closest to the tip of the probe (Bank 0). The probe’s ground and reference pads were shorted together and soldered to a gold pin, which was then connected to a gold pin that had been soldered to a skull screw on the animal. Recordings were made in external reference mode.

#### Probe insertion

On recording days, the probe was first dipped in one of three different colors of dye (DiI, DiD, and DiO). Different colors were used to allow up to three different penetrations in the same craniotomy to be distinguished in histology. The mouse was head-fixed on the VR rig and the KwikCast was removed from above the craniotomy. The probe was mounted on the rig at a 10-degree angle from vertical, lowered down to the level of the skull, aligned mediolaterally with the fiducial mark, and inserted as close to the transverse sinus as possible. The well was filled with saline (0.9% NaCl) and covered with a layer of silicone oil to prevent drying. The probe was inserted slowly (~10 um/s) until there was no longer any activity at the tip or until the probe started to bend. The probe was retracted 100–200 *μ*m from its deepest penetration point and left to settle for 30 min before starting the recording.

#### VR sessions

Recordings lasted between 40 and 150 min. The VR emitted a TTL pulse every frame from an Arduino UNO, and these pulses were recorded in SpikeGLX using an auxiliary National Instruments data acquisition card (NI PXIe-6341 with NI BNC-2110) to synchronize VR traces with neurophysiological data.

#### Probe cleaning

After each recording session, the probe was rinsed with deionized water, soaked in deionized water for at least 15 min, soaked in 2% Tergazyme for at least 60 min, and rinsed and soaked again in deionized water for at least 15 min.

#### Histology

After the last recording session, the mouse was killed with pentobarbital and perfused transcardially with 1X phosphate-buffered saline followed by 4% paraformaldehyde (PFA). Brains were extracted and stored in 4% PFA for at least 24 h after which they were transferred to 30% sucrose and stored for at least 48 h. Finally, brains were rapidly frozen and cut into 65 µm sagittal sections with a cryostat, stained with DAPI, and imaged with a widefield fluorescence microscope (Axio Imager 2, Zeiss).

#### Pupil tracking

We recorded video of the mouse’s face under IR illumination to track eye movements using a Guppy Pro camera (Allied Vision). Face camera frames were triggered once every three VR frames by TTL pulses from the VR, and these pulses were also recorded in SpikeGLX to synchronize the face videos with neurophysiological data.

To track the pupil in each camera frame, we first isolated the region of the frame that contained the eye. We then identified the pupil by manually selecting a pixel intensity threshold and finding the largest region of connected pixels in which all pixels were darker than the threshold. In order to prevent against the identification of a shadow instead of a pupil, we also manually defined a region over which the pupil was expected to be located and searched for connected pixels only within this region. If the number of connected pixels was in a sufficient range (>50 pixels, <1000 pixels), we then fit a circle to the connected pixels, and recorded the *x*- and *y*-coordinates of the center of the circle as the *x* and *y* location of the pupil relative to the coordinate frame of the camera. Coordinates of the pupil during time frames with poor tracking (as evidenced by inappropriate estimated pupil size) were estimated via interpolation.

Alongside pupil position, we tracked the corneal reflection (CR) resulting from the IR camera. As the location of the CR will move with camera jitter, we used the CR as a reference landmark from which pupil movements were computed. To track the CR, we set a pixel intensity threshold, set an expected CR boundary region, identified all connected pixels above this threshold within this region, and recorded the coordinates of the center of the best-fit circle to the connected pixels. We then computed horizontal and vertical deviations of the CR from its average location and subtracted these deviations from the pupil position.

To remove slow drift from the estimate of the pupil location, which may occur if the camera slowly changes position throughout the experiment, the data were high-pass filtered by subtracting a smoothed *x*- and *y*-position (smoothed with a 1000-point normalized Gaussian) from the original *x*- and *y*-position. We then removed extremal values, defined to be the top and bottom 1% of the position values. To convert pixel *x*- and *y*-coordinates, which are in the coordinate frame of the camera, to horizontal and vertical eye movements, we manually defined the horizontal and vertical axes of the pupil. The horizontal axis was defined as the line that connects the tear duct with the outermost region of the eye. The length of the line drawn was approximately equivalent to the width of the eye. The vertical line was orthogonal to the horizontal axis and centered on the average horizontal eye position. We projected the *x*- and *y*-values onto the eye-defined axes, divided the resulting values by the eye width (computed along the horizontal line), and used the output as the normalized horizontal and vertical position of the pupil. Thus, horizontal and vertical eye position (*E*_*h*_ and *E*_*v*_) are presented in units of fraction of the eye width, horizontal and vertical eye velocity ($\dot E_h$ and $\dot E_v$) are presented in units of fraction of the eye width/s. Both eye position and eye velocity were smoothed using Gaussian filters prior to analysis (using the “gausswin” function in MATLAB; position: Gaussian filter with *σ* ≈ 10 ms; velocity: Gaussian filter with *σ* ≈ 140 ms). Removal of extremal events (described below for all variables in the LN model framework), combined with smoothing, effectively removed saccades from the eye velocity data.

#### Spike sorting and synchronization

Spike sorting was performed offline using Kilosort2 (https://github.com/MouseLand/Kilosort2)^60^. Clusters were manually inspected and curated in Phy (version 2.0, https://github.com/cortex-lab/phy). Spike times and cluster identities were extracted from the output of KiloSort2 and Phy using code from the Spikes repository (https://github.com/cortex-lab/spikes). VR data (VR position and lick times) were synchronized to spiking data by substituting the time of each VR frame with the time of the corresponding detected TTL pulse. Synchronization was checked by comparing the difference between subsequent VR frame times with the difference between subsequent TTL pulse times and confirming that these were highly correlated (*P* > 0.95). Because the frame rate of the VR was not constant but instead fluctuated slightly around 60 Hz, we used linear interpolation to resample VR data to a constant 50 Hz for ease of subsequent analysis.

### LN model framework

#### Model formulation

We used a linear-nonlinear Poisson (LN) model^27^ to determine whether MEC neurons significantly encoded one or more behavioral variables. In the open field dataset, we examined variables pertaining to body and head movements: body position (*B*), body speed (*B*_*s*_), head direction about the azimuth axis (*H*_*a*_), angular head velocity about the azimuth axis ($\dot H_a$), head pitch angle (*H*_*p*_), angular head velocity about the pitch axis ($\dot H_p$), head roll angle (*H*_*r*_), and angular head velocity about the roll axis ($\dot H_r$). In the head-fixed dataset, we examined variables pertaining to body and eye movements: body position along the virtual reality track (*B*), body speed along the virtual reality track (equivalent to body speed on the running wheel; *B*_*s*_), horizontal eye position (*E*_*h*_), vertical eye position (*E*_*v*_), horizontal eye velocity ($\dot E_h$) and vertical eye velocity ($\dot E_v$).

These models quantify the dependence of spiking on one or more variables by estimating the spike rate ($\hat r_t$) of a neuron during time bin *t* as an exponential function of a sum of model inputs (See Supplementary Fig. 2). These inputs include a time-invariant value that sets the baseline firing rate (*b*_0_, learned through model fitting), and a time-varying input derived from the learned contribution of each variable at time *t* to $\hat r_t$. For each variable, this input is computed as the dot product of an “animal state” vector, which denotes the variable value at time *t* for each variable (see below), with a corresponding set of parameters learned by the model. $\hat r_t$ is concatenated to form $\hat r$, a vector of firing rates for one neuron over *T* time points. This quantity is expressed mathematically as

8 $$\begin{array}{*{20}{c}} {\hat {\mathbf{r}} = \frac{{{\mathrm{exp}}\left( {b_0 + \mathop {\sum }\nolimits_i X_i^{\rm{d}}{\mathbf{w}}_{\mathbf{i}}} \right)}}{{{\rm{d}}t}}} \end{array}$$

where *i* indexes the variable (for example, in the freely moving dataset, $i \in \{ B,B_s,H_a,H_p,H_r,\dot H_a,\dot H_p,\dot H_r\}$), *X*_*i*_ is a *W*_*i*_ × *T* matrix where each column is an animal-state vector (**x**_**i**_, length *W*_*i*_) for variable *i* at time bin *t*, **w**_**i**_ is a *W*_*i*_ × 1 column vector of learned parameters that converts animal state vectors into a firing rate contribution, and d*t* is the time bin length (20 ms).

Each value in an animal-state vector denotes a binned variable value. For the open field dataset, we used the length/width of the arena (in cm) × 15/100 as the number of position bins, 10 azimuth head direction bins, speed bins with width of 5 cm/s, and 8 bins for pitch, roll, and azimuthal head velocity. For the virtual reality dataset, we used 20 position bins, speed bins with width of 5 cm/s, and 5 bins for all eye-related variables. Elements of this vector were computed through cardinal spline interpolation following procedures similar to^61^. Briefly, at each time point *t*, the fractional distance *a*^(*t*)^ was computed for each current variable value (e.g. body position) to the nearest binned values (e.g. pre-determined position bins). Specifically, *a*(*t*) = $\frac{{y^{(t)} - z_j}}{{z_{j + 1} - z_j}}$, where *y*^(*t*)^ is the current variable value, and *z*_*j*_ and *z*_*j*+1_ are the nearest binned values to the left and right, respectively. This value was then used to compute the animal state vector values that correspond to the parameters for the $j - 1,j,j + 1,$ and *j* + 2 bins:

9 $$\begin{array}{*{20}{c}} {\left[ {\begin{array}{*{20}{c}} {X_{i,j - 1}^{\left( t \right)}} & {X_{i,j}^{\left( t \right)}} & {X_{i,j + 1}^{\left( t \right)}} & {X_{i,j + 2}^{\left( t \right)}} \end{array}} \right] = \left[ {\alpha \left( t \right)^3\alpha \left( t \right)^2\alpha \left( t \right)1} \right]\left[ {\begin{array}{*{20}{c}} {-s} & {2 - s} & {s - 2} & s \\ {2s} & {s - 3} & {3 - 2s} & { - s} \\ { - s} & 0 & s & 0 \\ 0 & 1 & 0 & 0 \end{array}} \right]} \end{array}$$

We used *s* = 0.5–0.7 but found our results to be largely insensitive to this parameter.

#### Model fitting and performance

To learn the variable parameters *w*_*i*_ and *b*_0_, we used MATLAB’s fminunc function to maximize the Poisson log-likelihood of the observed spiketrain (*n*) given the model spike number ($\hat r * dt$) and under the prior knowledge that the parameters should be small through an L2 penalty (with hyperparameter *β* = 1). Model performance for each cell was computed as the increase in log-likelihood of the model compared to the log-likelihood of a mean firing rate model. The performance was quantified through 10-fold cross-validation, where each fold was 10% of the data and chosen from 10-s sections of the entire dataset such than no two folds overlapped. To identify which variable, or set of variables, a neuron encoded, a heuristic forward-search algorithm was employed that determines whether adding variables significantly improves model performance (*P* < 0.05 for a one-sided signed rank test^27^). In this procedure, for each cell we first fit all variables individually, and determined which variable was most predictive of neural spiking on held-out data. We then fit all possible double-variable models that included the original most predictive variable and determined if the best-performing double-variable model significantly outperformed the single-variable model. Differences in performance were assessed via a one-sided Wilcoxon signed-rank test, with α = 0.05. If the double-variable model significantly outperformed the single-variable model, we then re-fit all possible triple-variable models that contained the variables within the double-variable model. This procedure continued until adding an additional variable did not significantly increase held-out model performance. At the end of the forward-search procedure, we determined whether the selected model performed significantly better than a baseline, mean firing rate model. If so, the cell was classified as significantly encoding each of the variables in the final model; otherwise, the cell was classified as not significantly encoding any variable.

#### Data pre-processing

To ensure that the spikes used to train the LN model occurred during adequately sampled behavioral states, for each session we first computed the behavioral distribution for each variable, and limited the model input to epochs that occurred when the animal’s behavior fell between either the 2.5th and 97.5th percentiles (i.e., the inner 95% of the behavioral distribution) for open field data, or between the 1st and 99th percentiles (i.e., the inner 98% of the behavioral distribution) for head-fixed data. We used the parameters learned by the LN model to derive tuning curves for each variable significantly encoded. Additional details of the model framework used here are described in Hardcastle et al.^27^.

#### Computing contribution to model performance

To compute the contribution of a variable to the model performance for a single cell, we computed the difference between the model performance (log-likelihood increase) of the selected model (i.e., the model containing all significantly encoded variables) and a reduced model in which the variable of interest was removed. This quantity, which represents the contribution of that variable to the model performance, was then normalized by the model performance for the selected model and subtracted from 1.

Additionally, we performed this procedure using correlation coefficients as the measure of model performance. In this case, the spiketrain was smoothed with a Gaussian filter using the MATLAB “gausswin” function, using a 20-bin window, and correlated with the estimated firing rate as computed by the selected or reduced model.

#### Model-derived tuning curves

Model-derived tuning curves were constructed for each variable using the parameters learned by the LN model. These tuning curves return an output similar to standard firing rate based tuning curves, with the exception that the average firing rate for a given variable value (e.g. a certain position) has the contribution of other variables regressed out. Overall, we found the model-derived and firing rate-derived tuning curves for a given variable to be highly correlated (see Supplementary Fig. 5). To compute the tuning curve (*C*) for a variable *i*, we first binned values taken by variable *i* into *M* bins (indexed by *m*; default *M* = 200). We then computed the expected firing rate for each bin using the learned parameters **w**_**i**_, a feature vector ${\mathbf{Y}}_{\mathbf{i}}^{({\mathrm{m}})}$ that returns the “animal-state” vector assuming that variable *i* takes the value within bin *m*, and scaling variable *γ* that denotes the average firing rate contribution from all other variables that are significantly encoded by that cell:

10 $$\begin{array}{*{20}{c}} {C_i\left( m \right) = \exp \left( {{\mathbf{Y}}_i^{\left( m \right)} \ast {\mathbf{w}}_{\mathbf{i}}} \right) \ast \frac{\gamma }{{dt}}} \end{array}$$

The value of *γ* is computed by $\gamma = \exp \left( {b_0} \right) \ast \prod _{v,v \ne i}\left( {\frac{1}{T}\mathop {\sum}\nolimits_{{t}} {\exp } ({\mathbf{X}}_{\mathbf{v}}^{({\mathbf{t}})} \ast {\mathbf{w}}_{\mathbf{v}})} \right)$, which is the baseline firing rate multiplied by the average firing rate contribution from every other variable (indexed with *v*).

#### Identifying rate of falsely detected encoded variables

To verify that the LN model identified meaningful relationships between a navigational variable and a cell’s spiketrain, we estimated the rate at which each variable was falsely selected as being significantly encoded by a cell. We performed this analysis for Cohort 1 of the freely moving dataset, and the entire head-fixed VR dataset. For each variable in each dataset, we randomly shuffled the behavioral values across the time axis. Note that to de-couple spiking and behavioral data, one can shuffle spikes relative to behavior, or behavior relative to spikes. We chose the latter, as this method allowed us to shuffle each variable independently and thus identify the false-positive rate for each variable independently. For example, in one iteration, we randomly shuffled all body position values. We then re-ran the entire model-selection and model-fitting procedure on the dataset, and recorded which variables were significantly encoded for each cell. We used this approach to determine how frequently each variable of interest (in this example, position) was falsely detected as significant using the LN model (see Supplementary Fig. 3).

#### Generating simulated data without eye-encoded information

To determine whether the LN modeling framework falsely identified eye movement-related tuning due to correlations with eye position or eye velocity and body position or speed on the VR track, we generated a simulated dataset that was identical to the original, but without eye movement-related information (see Supplementary Fig. 12). To accomplish this, we first identified the selected variables of a given cell (i.e. by fitting a series of LN models) and identified the model parameters corresponding to each variable. We then generated a new spiketrain using all parameters except those relating to eye movements. Specifically, we considered the estimate of the cell’s firing rate over T time points, $\hat r = {\mathrm{exp}}(A * w)$, where *A* is a matrix of navigational information across all time points and concatenated horizontally across all encoded variables, and *w* is a stacked vector of parameters across all encoded variables. By deleting the relevant entries in *A* and *w* that correspond to eye movement information, we generated a new $\hat r$, and drew from a Poisson distribution with mean given by each element in $\hat r$ to generate a new spiketrain. We then ran the model-fitting and model-selection procedure again on the newly generated spiketrain and recorded the set of encoded variables.

#### Comparison between LN and shuffling-based methods for determining statistical significance

The LN model offers several advantages over commonly used shuffling-based methods to determine whether a neuron significantly encodes a behavioral variable. In particular, the LN model approach does not require a pre-determined tuning curve shape (e.g., linear), and is able to account for the contributions of correlated behavioral variables. However, we further verified our findings using two common shuffling-based methods: within-cell shuffling^19^, and pooled shuffling^30^ (See Supplementary Fig. 4). In both methods, a modulation score was computed for each cell and then compared to either a shuffled distribution of modulation scores obtained by shifting the individual cell’s spiketrain in time (within-cell shuffling) or a shuffled distribution of modulation scores obtained by shifting the spiketrains of all cells (pooled shuffling). We employed both approaches for the analyses below.

First, we identified cells linearly encoding a head movement variable (*H*_*p*_, *H*_*r*_, or $\dot H_a$) in the open field dataset or an eye movement variable (*E*_*h*_, *E*_*v*_, $\dot E_h$, or $\dot E_v$) in the head-fixed VR dataset. For each cell, the Pearson’s correlation coefficient between instantaneous behavior (i.e., pitch position) and firing rate was computed. To generate shuffled distributions, each cell’s spiketrain was shifted by a random amount of time exceeding 20 s and the modulation score was recomputed. 500 shuffles were generated for each cell. A cell was considered significant if its correlation coefficient fell either below the 2.5th percentile or above the 97.5th percentile of the shuffled distribution of correlation coefficients (for within-cell shuffling, these percentiles were determined from the individual cell’s shuffled distribution; for the pooled shuffling they were determined from the shuffled distribution of all cells combined).

Using the LN model approach, we observed many cells with nonlinear tuning. For example, we observed “nonlinear symmetric” head velocity and eye velocity cells that increased or decreased their firing rate with increasing head or eye speed in either direction^29^; these cells thus had V-shaped- or inverse-V-shaped-tuning curves. To identify symmetric cells via shuffling methods, we separately computed the correlation coefficients between velocity and firing rate for velocities below zero and above zero (corresponding to rightward versus leftward head rotations, temporal versus nasal eye rotations in the horizontal plane, and downward versus upward velocities eye rotations in the vertical plane). A cell was considered to be significantly symmetrically tuned to head or eye velocity if either: 1) its correlation coefficient below zero fell below the 5th percentile of the shuffled distribution and its correlation coefficient above zero fell below above the 95^th^ percentile of the shuffled distribution (identifying V-shaped tuning) or 2) its correlation coefficient below zero fell above the 95th percentile of the shuffled distribution and its correlation coefficient above zero fell below the 5th percentile of the shuffled distribution (identifying inverse-V-shaped tuning). We then compared the number of cells identified as significantly encoding each variable using the LN approach and the shuffling approaches (see Supplementary Fig. 4 for further details).

### Tuning curves

#### Raw tuning curves

Raw tuning curves, presented in Supplementary Figs 5, 6, were constructed for each variable by binning the behavioral variable into 20 bins, and dividing the total number of spikes emitted in each bin by the animal’s occupancy in that bin. The behavioral range considered was determined as in Visualizing tuning curves. Raw tuning curves were smoothed using Matlab’s rlowess function (smoothing over 7 bins).

#### Visualizing tuning curves

To visualize model-derived or spike-derived tuning curves, lower and upper limits were selected in the following manner: For azimuth head direction, the lower and upper limits were 0 and 360°, respectively. For all other head movement variables, the lower limit was the greater value between: (1) the 2.5^th^ percentile of the behavioral distribution and (2) a preset lower bound [−50° for *H*_*p*_ or *H*_*r*_; −100°/s for $\dot H_a$]; the upper limit was the lesser value between (1) the 97.5^th^ percentile of the behavioral distribution and, (2) a preset upper bound [50° for *H*_*p*_ or *H*_*r*_; 100°/s for $\dot H_a$]. For eye movement variables, the lower limit was the greater value between: (1) the 1st percentile of the behavioral distribution and, (2) a preset lower bound [−3 eye units for *E*_*h*_; −2 eye units for *E*_*v*_; −3 eye units/s for $\dot E_h$; −2 eye units/s for $\dot E_v$]; the upper limit was the lesser value between (1) the 99^th^ percentile of the behavioral distribution and (2) a preset upper bound [3 eye units for *E*_*h*_; 2 eye units for *E*_*v*_; 3 eye units/s for $\dot E_h$; 2 eye units/s for $\dot E_v$].

#### Calculating tuning curve stability

The stability of tuning to a given variable was quantified as the Pearson’s correlation coefficient between model-derived tuning curves generated separately for the first and second halves of a session. In generating separate tuning curves, only the behavioral epochs falling within the inner 95th or 98th percent of the range (for freely moving and head-fixed data, respectively) were used. In each half, we fit an LN model using the variables that were determined to be significantly encoded over the full-length session. Prior to computing stability, tuning curves were restricted to the behavioral range described in Visualizing tuning curves. Additionally, the two tuning curves were restricted to the portion for which the behavioral ranges overlapped. If the two tuning curves then contained an unequal number of datapoints, the shorter tuning curve was up-sampled to match the number of datapoints in the longer tuning curve. Raw tuning stability (shown in Supplementary Fig. 6) was computed in the same manner, except the raw tuning curves for each half of the session were compared.

#### Quantifying tuning curve shape

Cells encoding head or eye movements were classified into subcategories according to the shape of their model-derived tuning curves. For each variable, we restricted analyses to a limited range (see Visualizing tuning curves). The shape of a tuning curve was classified in the manner below only if the animal explored at least 50% of this range (for head-related variables) or 80% of this range (for eye movement-related variables).

Tuning curve shapes were categorized using a greedy forward-selection model search procedure using polynomials of increasing complexity. Tuning curves were first range-normalized to be between 0 and 1. Tuning curves for *H*_*p*_ and *H*_*r*_ were also smoothed with a Gaussian filter (using the MATLAB function “smoothdata” function with a width = 20). We then fit a series of polynomials of increasing complexity, from a linear fit (1st degree polynomial) up to a 5th-degree polynomial, to each tuning curve. We began with a linear fit, and computed the mean squared error alongside the fraction of error explained compared to a baseline 0-degree polynomial (i.e. a flat-line average):

11 $$\begin{array}{*{20}{c}} {{\rm{Fraction}}\,{\rm{error}}\,{\rm{explained}} = \frac{{{\rm{MSE}}\left( {{\rm{baseline}}\,{\rm{model}}} \right) - {\rm{MSE}}\left( {{\rm{polynomial}}\,{\rm{model}}} \right)}}{{{\rm{MSE}}\left( {{\rm{baseline}}\,{\rm{model}}} \right)}}} \end{array}$$

If the fraction of error explained was greater than 0.9, we then recorded the current model as the selected model. Otherwise, we then fit a polynomial of one higher order, and repeated the MSE comparison.

#### Quantifying head and eye velocity tuning curves

To identify the location of the peak or valley in a tuning curve, we used the “findpeaks” function in MATLAB to identify peaks and valleys (using the negative of the signal). We then recorded peaks and valleys that took the maximum or minimum of the signal. To examine the shape of the velocity tuning curves, we also analyzed fit a line to the tuning curve on either side of the origin (0 velocity).

### Entropy and mutual information

#### Entropy of data-generated distributions and uniform distributions

We computed the entropy *H* of a distribution of *X* as

12 $$\begin{array}{*{20}{c}} {H\left( x \right) = - \mathop {\sum }\limits_i P\left( {X_i} \right)\log \left( {P\left( {X_i} \right)} \right)} \end{array}$$

where *X* is binned, and *P*(*X*_*i*_) = the probability that a randomly drawn value from *X* occurs within the boundaries defined by bin *i*. We computed the entropy of a uniform distribution by generating a dataset equivalent in size to *X* under the assumption that the number of occurrences in each bin were equal, and re-computing the entropy.

#### Computing mutual information between eye, position, and speed variables

To compute the mutual information *I* between two continuous variables A and B, we first binned both variables, and then computed:

13 $$\begin{array}{*{20}{c}} {I\left( {A,B} \right) = \mathop {\sum }\limits_i \mathop {\sum }\limits_j P\left( {A_i,B_j} \right) \ast \log \left( {\frac{{P\left( {A_i,B_j} \right)}}{{P\left( {A_i} \right)P\left( {B_j} \right)}}} \right)} \end{array}$$

Indexes in each sum refer to individual bins, and probabilities were estimated empirically from the data (i.e. *P*(*A*_*i*_) = the probability that variable A occurs within the boundaries defined by bin *i*). Mutual information on shuffled data (computed to generate a null distribution with which to compare the data-derived mutual information) was computed by shuffling a variable, and re-computing the mutual information.

### Using PCA to identify neural clusters

To compare all tuning curves of a given variable across cells (Fig. 3g, h and Supplementary Fig. 11), we built an *n* × *m* matrix *X* for each set of curves, where *n* = number of cells in the analysis and *m* = number of bins in the tuning curve. For example, since there are 46 cells that encode pitch and 50 tuning curve bins, the corresponding *X* matrix is 46 × 50. Rows of X were mean-subtracted and re-scaled to have unit range. In order to visualize each cell’s location in a “tuning curve space”, in which each axis is defined by a tuning curve bin (thus returning a 50-dimensional space for pitch tuning curves), we projected these data onto a two-dimensional subspace using principal component analysis (PCA). Neighboring cells in this space exhibit similar tuning curves.

To determine whether cells with similar tuning curves for a given variable *i* encoded other similar variables, we generated a matrix *X* for variable *i* and plotted cells in the associated tuning curve space. For each additional variable (all other variables that were not variable *i*), we labeled all points according to whether the cell also encoded this variable and looked for clustering amongst the labeled datapoints. Clustering was determined by computing the average distance (*d*) to each neuron’s *k*-nearest neighbors of the same type (i.e. those that encoded the second variable). The value of *k* was varied from 1–10. We chose 10 as the maximum as some sub-populations had small numbers of cells (e.g., there were 5 cells encoding azimuthal head velocity and body position; in this case *k* = 5 and *k* = 10 are equivalent). The likelihood of observing *d* was then compared to a null distribution. This distribution was generated by scrambling the datapoint labels and re-computing mean nearest-neighbor distances. *P* values were computed by analyzing the fraction of mean nearest-neighbor distances that were smaller than what was observed in the data. The resulting *p* value is Bonferroni corrected, with significance being achieved by *P* = 0.05/10.

### Reporting summary

Further information on research design is available in the Nature Research Reporting Summary linked to this article.

## Supplementary information

Supplementary Information

Peer Review File

Reporting Summary

## Data Availability

The raw data used in all analyses within this manuscript are available at https://figshare.com/authors/Lisa_Giocomo/9864194.

## Code availability

Custom MATLAB code generated to fit the series of linear-nonlinear-Poisson (LN) models can be found at: https://github.com/GiocomoLab/ln-model-of-mec-neurons and https://github.com/khardcastle/spline-lnp-model.
