# Supplementary material for: Mouse entorhinal cortex encodes a diverse repertoire of self-motion signals
Source: Nat Commun. 2021 Jan 28;12:671. doi: 10.1038/s41467-021-20936-8 (PMC7844029; doi:10.1038/s41467-021-20936-8)
Supplement: Supplementary file 1 — Supplementary Information [file 41467_2021_20936_MOESM1_ESM.pdf]

Supplementary Fig 1

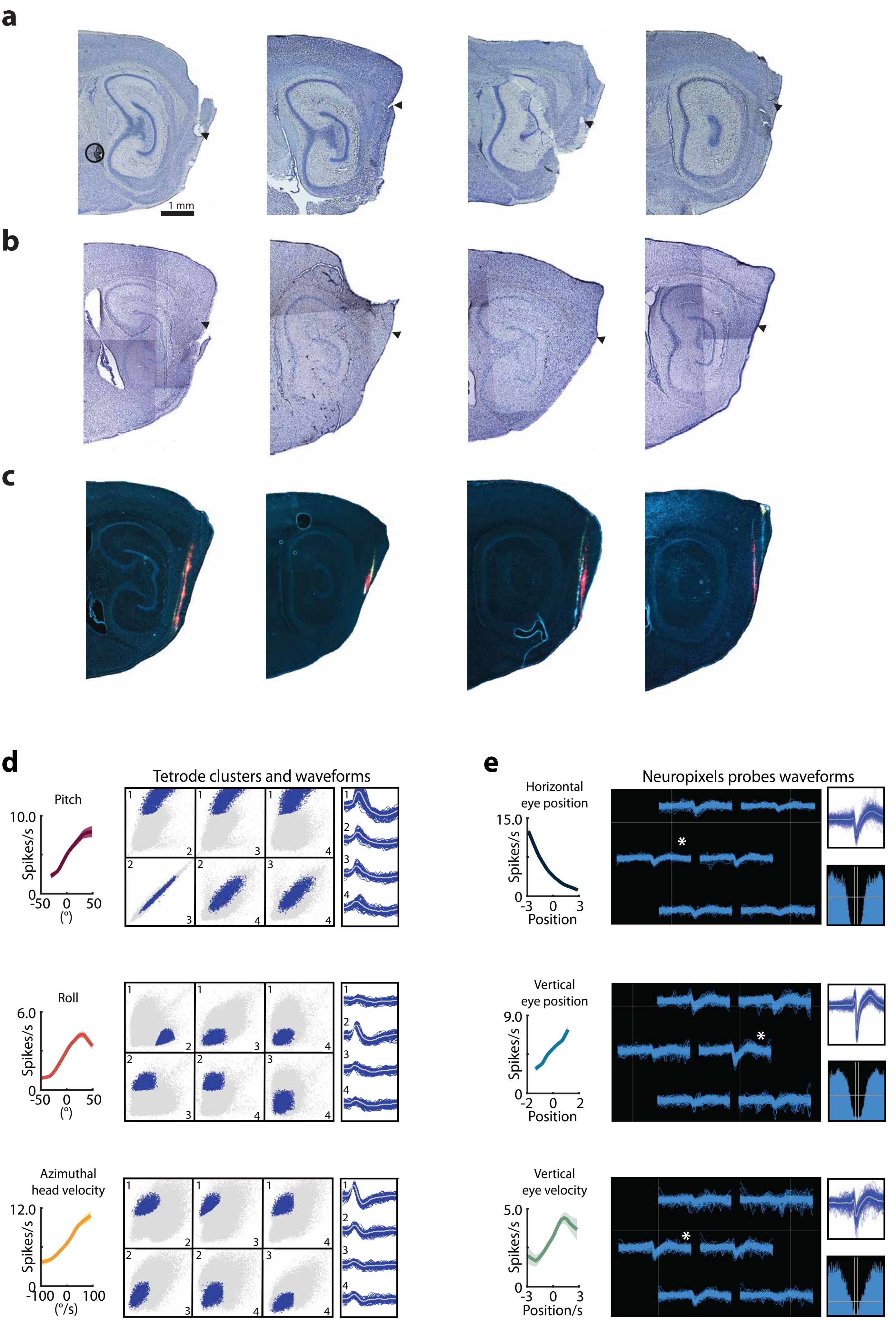

Supplementary Figure 1. Histology for representative animals from each cohort in the study and example waveforms. **a-b,** Nissl stained brain sections showing the final tetrode placements in four mice from the freely-moving datasets without **(a)** and with **(b)** accelerometers sensors. Black arrows mark the final tetrode placement. Tetrodes placements were similarly confirmed in the remaining 22 tetrode-implanted mice. **c**, Dapi stained brain sections showing the placement of the Neuropixels probe (red, yellow or blue, DiD, DiI, DiO,), in four mice used for head-fixed eye tracking experiments. Neuropixels probe placements were similarly confirmed in the remaining 4 Neuropixels probes-implanted mice. **d***,* Left: Example raw tuning curves for cells encoding pitch, roll, or azimuthal head velocity. Right: The cells' action potential waveforms, recorded via tetrodes. The amplitude of all spikes recorded on the tetrode (gray) is compared on pairs of channels (labeled 1–4). Spikes emitted from the example cell are clustered in blue. Far right: the cell's action potential waveforms on each channel (blue), with the mean overlaid (gray). **e**, Left: Example raw tuning curves for cells encoding horizontal eye position, vertical eye position or vertical eye velocity. Right: The correspeonding cells' action potential waveforms. For the head-fixed dataset (horizontal and vertical eye position and velocity), cells were recorded using Neuropixels probes. Traces at left show the action potential waveforms recorded on neighboring probe channels; astericks indicate the channels on which the waveforms had the largest amplitude. Far right: zoomed in view of the action potentials (blue traces) recorded on the channel with the largest amplitude; the average waveform is shown in gray. Bottom far right; autocorrelogram of the spike train. Vertical gray lines denote +/- 2 ms. Horizontal gray line shows the baseline firing rate. Only cells with clear refractory periods were included in the dataset.

Supplementary Fig 2

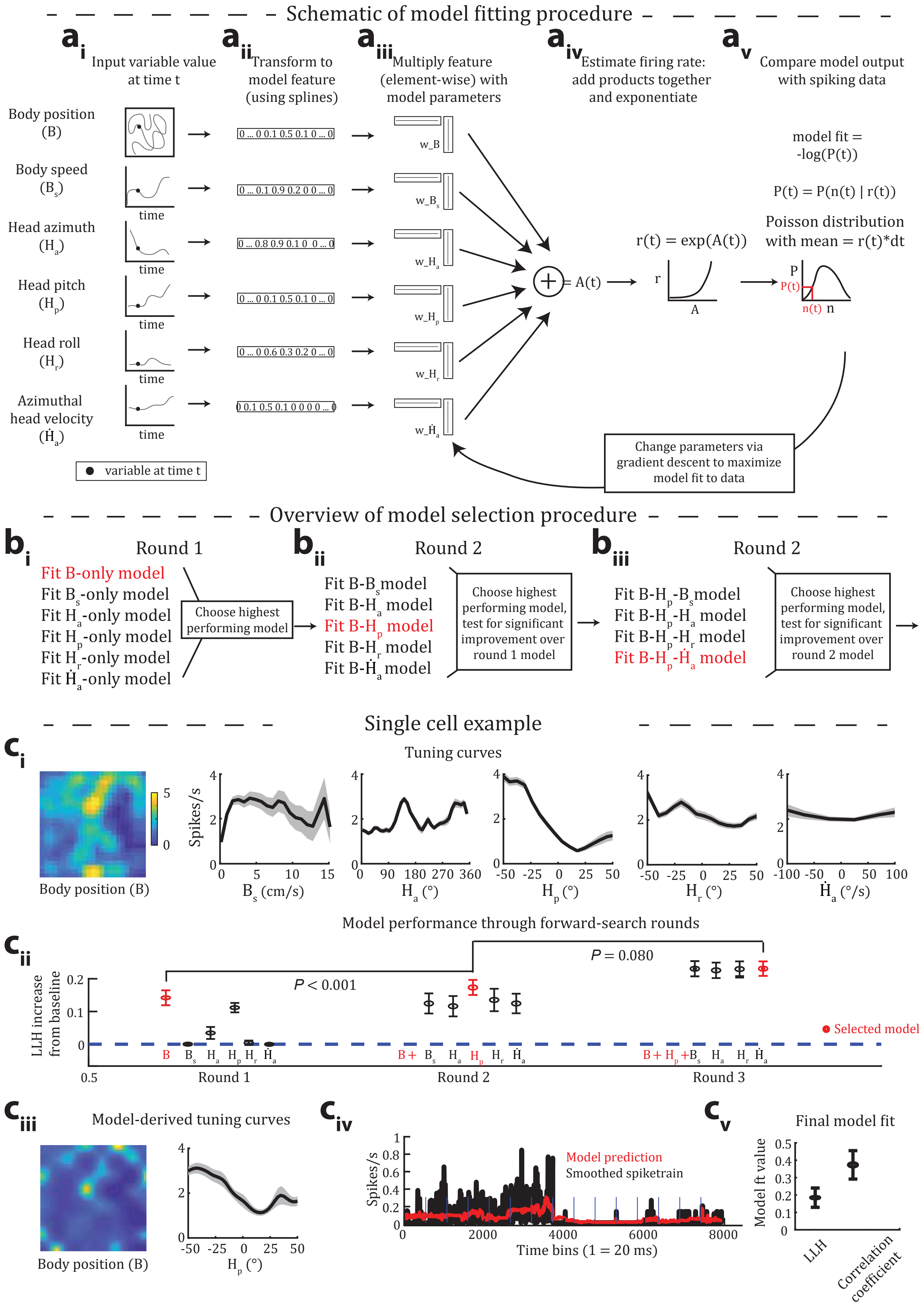

Supplementary Figure 2: Description of LN model fitting procedure. **a**$_{\mathrm{i}}$, Schematic of procedure. The model receives input corresponding to animal state at time t. **a**$_{\mathrm{ii}}$, Input for each variable i is transformed into a vector $\mathbf{X_i}(t)$, where the magnitude of elements in $\mathbf{X_i}(t)$ correspond to the proximity of the variable value to each control point location (see Methods). **a**$_{\mathrm{iii}}$. The spline function models each variable's contribution to the natural log of the firing rate. **a**$_{\mathrm{iv}}$, To estimate the firing rate $r(t)$, inputs are summed and exponentiated. **a**$_{\mathrm{v}}$, We compare $r(t) * dt$ to the number of spikes, $n(t)$, observed during time t by computing the negative log probability of observing $n(t)$ spikes under a Poisson distribution with mean rate $r(t) * dt$. **b**$_{\mathrm{i}}$, To determine the minimal set of variables encoded, we use a forward-search feature-selection procedure. In the first round, we fit all single-variable models and identify the highest performing model. **b**$_{\mathrm{ii}}$, We then proceed to the second round, where we fit all double-variable models that include the first variable (here, B). We then find the best double-variable model (B-H_p), and determine whether adding the second variable (H_p) significantly improves model fit via a one-sided signed-rank test (no correction for multiple comparisons; here, *P* = 1e-16). **b**$_{\mathrm{iii}}$**,** If so, we fit all triple-variable models that include all previous variables. This procedure continues until there is no significant improvement. **c**$_{\mathrm{i}}$**,** Example tuning curves. Gray corresponds to the standard error of the mean. **c**$_{\mathrm{ii}}$, Results of the forward-search procedure. Error bars represent mean ± sem of the model fit across held out data (fold n = 10). Red bars indicate the selected model for that round. **c**$_{\mathrm{iii}}$, Model-derived tuning curves for variables significantly encoded. **c**$_{\mathrm{iv}}$, Estimated firing rate compared to the true firing rate. Blue lines indicate boundaries between chunks of data. The spike train is smoothed with a Gaussian filter, sigma = 200 ms. **c**$_{\mathrm{v}}$**,** Model fit in terms of log-likelihood increase from a mean firing rate model divided by the number of spikes (left), or correlation coefficient (over n = 10 data folds, error bars = mean ± sem).

Supplementary Fig 3

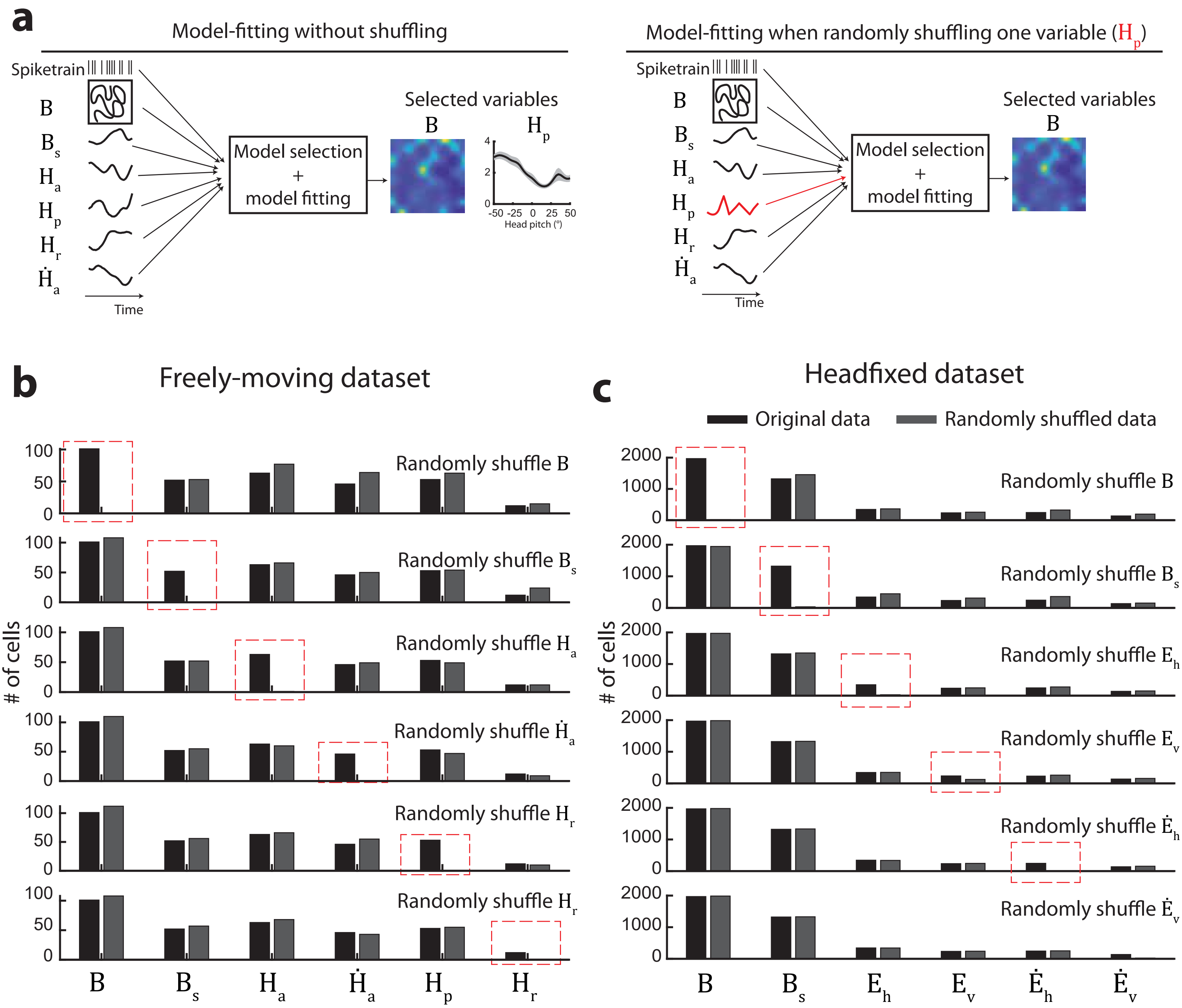

Supplementary Figure 3. Identifying the rate of false positives in the LN-model. In order to identify the rate of false positives in detection of an encoded variable, we randomly shuffled the values of that variable, re-fit the model, and re-identified the significantly encoded variables. **a,** Schematic of the LN model-selection and model-fitting process with original data (left), and with randomly shuffled data (right, for pitch). **b**, Comparison between the number of cells encoding each variable in the original dataset (black bars), with the number of cells detected as encoding each variable in a dataset in which a single variable was randomly shuffled (gray bars). The variable that was randomly shuffled is denoted in the upper right of each row. Dotted red rectangles highlight the relevant comparison. Note that in all cases, there was a relatively low chance of false positives (B: 0 falsely-detected/101 normally-detected; B_s: 1 falsely-detected/52 normally-detected; H_a: 0 falsely-detected/63 normally-detected; H_p: 0 falsely-detected/46 normally-detected; H_r: 0 falsely-detected/53 normally-detected; $\dot{H}_a$: 1 falsely-detected/12 normally-detected). **c**, Same as *b*, but for the dataset collected during head-fixation and virtual-reality navigation. Here we investigated the rate of false positives for B, B_s, E_h, E_v, $\dot{E}_h$, and $\dot{E}_v$. Here, we observed a very low chance of false positives for each variable (B: 0 falsely-detected/1958 normally-detected; B_s: 2 falsely-detected/1321 normally-detected; E_h: 5 falsely-detected/343 normally-detected; E_v: 5 falsely-detected/234 normally-detected; $\dot{E}_h$: 8 falsely-detected/244 normally-detected; $\dot{E}_v$: 13 falsely-detected/135 normally-detected).

Supplementary Fig 4

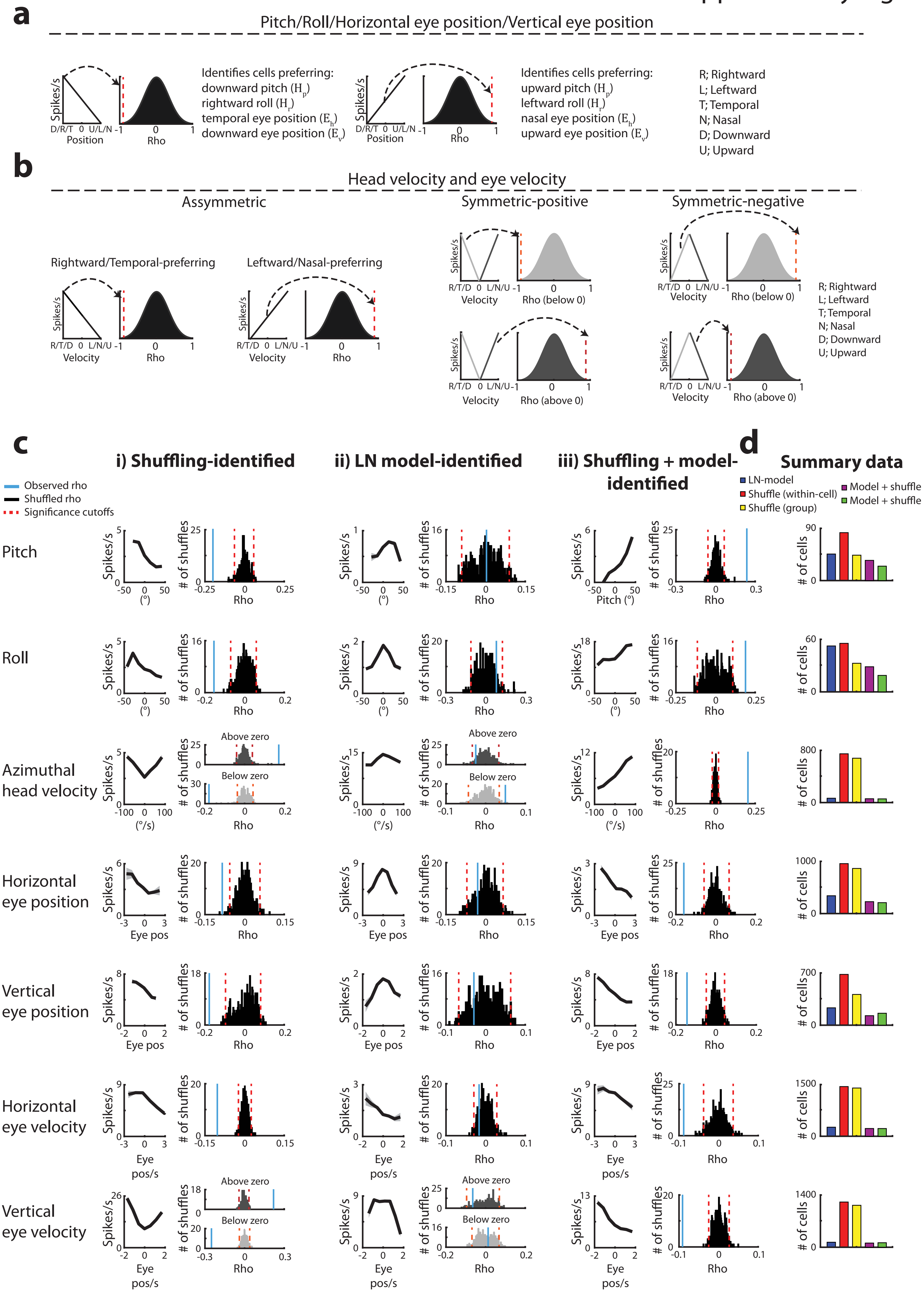

Supplementary Figure 4. Comparison between LN-model and shuffle identification of cells encoding head or eye movement. **a,** Cartoon illustrating the shuffling procedure to identify cells linearly encoding pitch, roll, horizontal eye position, or vertical eye position. We observed similar results using either shuffling method (panel d). Red dashed lines indicate the significance cutoffs. **b**, Cartoon illustrating the shuffling procedure to identify cells non-linearly encoding head velocity or eye velocity (see Methods for details). **c**, Example cells identified by i) shuffling but not the LN-model, ii) LN-model but not shuffling, iii) both shuffling and the LN-model. Each example shows the raw tuning curve at left, and the shuffled distribution of rho values obtained from within-cell shuffling at right. The observed Pearson's correlation coefficient (rho) is shown in blue and the significance cutoffs are shown in red. **d**, The number of cells identified by the LN-model (blue), within-cell-shuffling (red), pooled-shuffling (yellow), both the LN-model and within-cell shuffling (purple), or both the LN-model and pooled-shuffling (green). First, note that far more cells were identified by either the within-cell-shuffling or grouped-shuffling methods compared to the LN-model. Shuffling is more likely to yield false positives because it cannot account for correlations between behavioral variables. Second, note that nearly all of the cells identified by the LN-model were also identified by shuffling. Together, these additional statistical tests indicate that LN-model provides a conservative estimate of the number of cells significantly encoding each variable.

Supplementary Fig 5

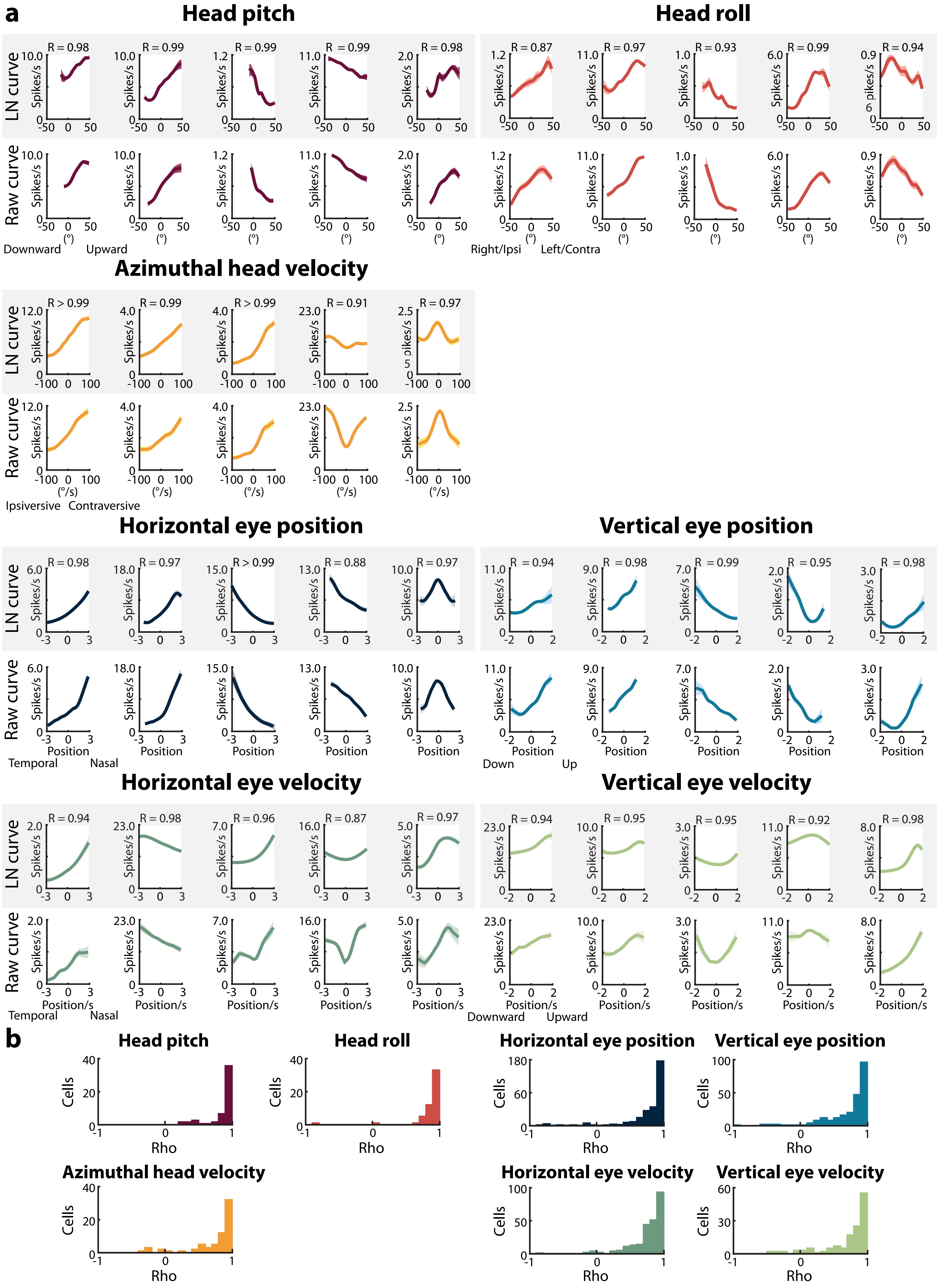

Supplementary Figure 5. Comparison between model-derived and raw tuning curves. **a**, Top rows (gray background)*:* Model-derived tuning curves for example cells. Bottom rows: The raw (spike-derived) tuning curves for the same cells (mean ± sem firing rate in each bin). The Pearson's correlation coefficient between the two curves is indicated at top ('R'). **b**, Histograms showing the correlation coefficient between the LN-model derived tuning curves and raw tuning curves for all cells (median [1st–3rd quartile] correlation coefficient: pitch = 0.94 [0.91–0.98], n = 46 cells; roll = 0.93 [0.88–0.96], n = 53 cells; azimuthal head velocity = 0.90 [0.69–0.97], n = 67 cells; horizontal eye position = 0.90 [0.73–0.96], n = 343 cells; vertical eye position = 0.85 [0.63–0.95], n = 234 cells; horizontal eye velocity = 0.85 [0.71–0.93], n = 244 cells; vertical eye velocity = 0.87 [0.71–0.93], n = 134 cells).

Supplementary Fig 6

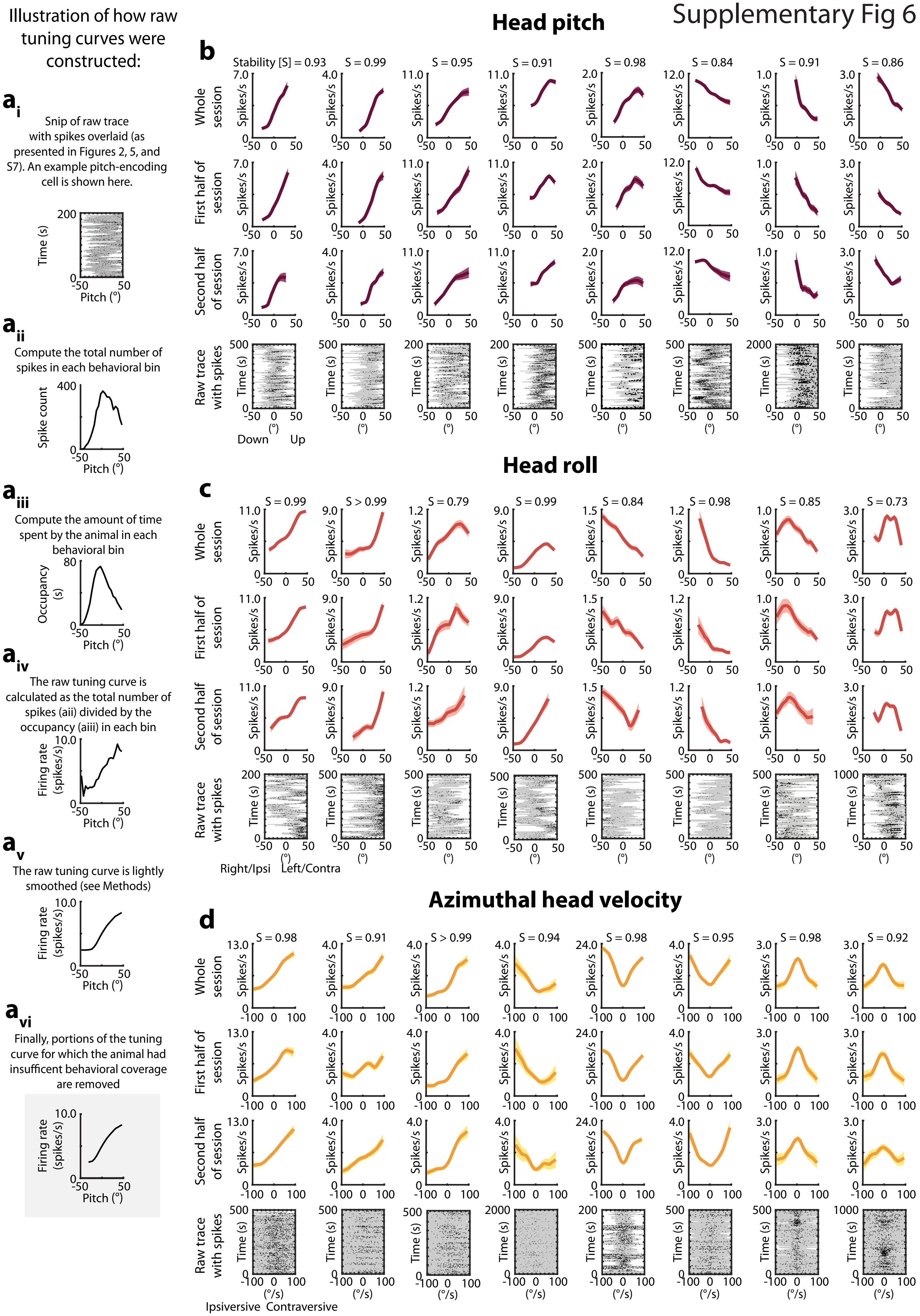

Supplementary Fig 6

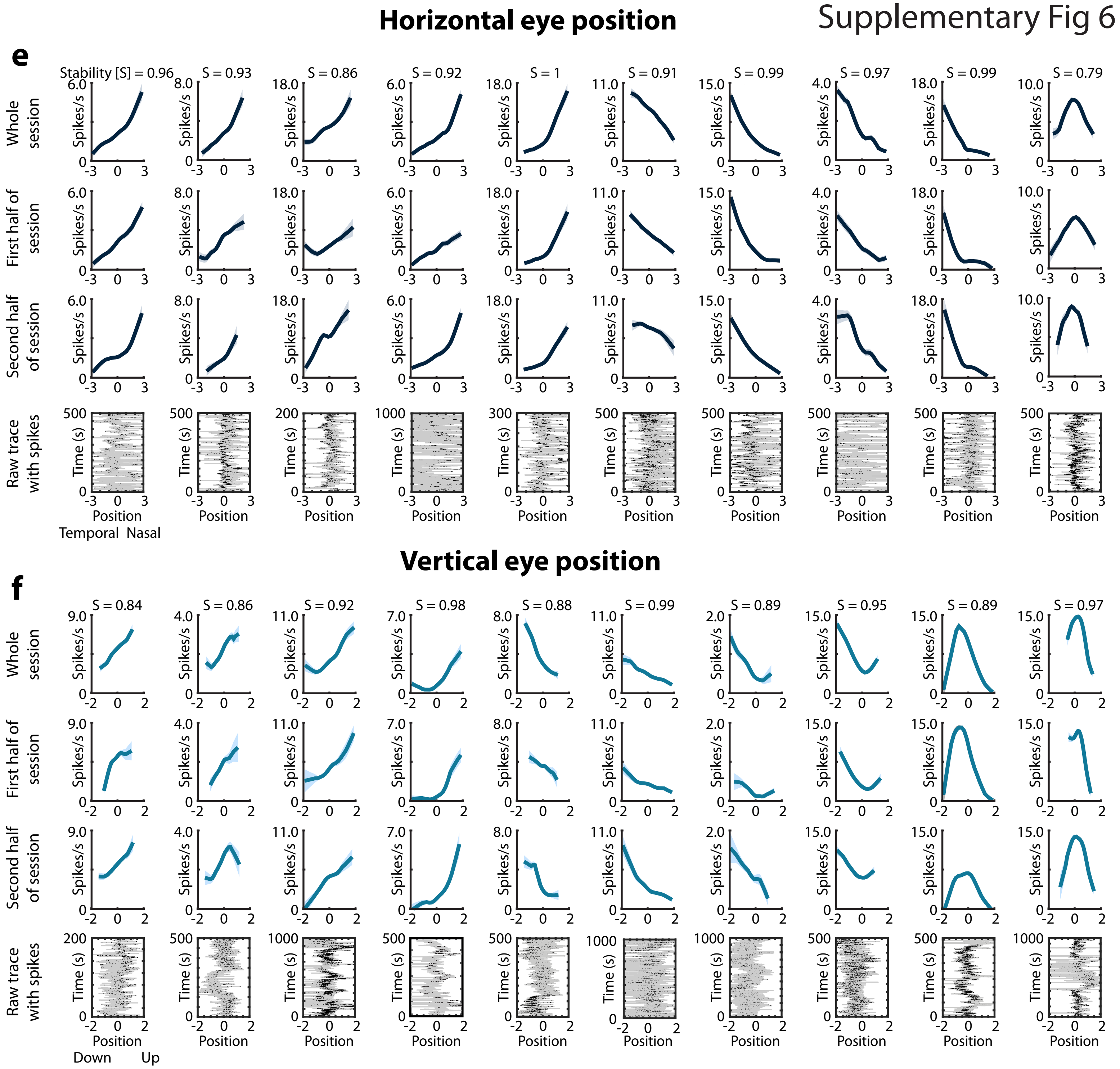

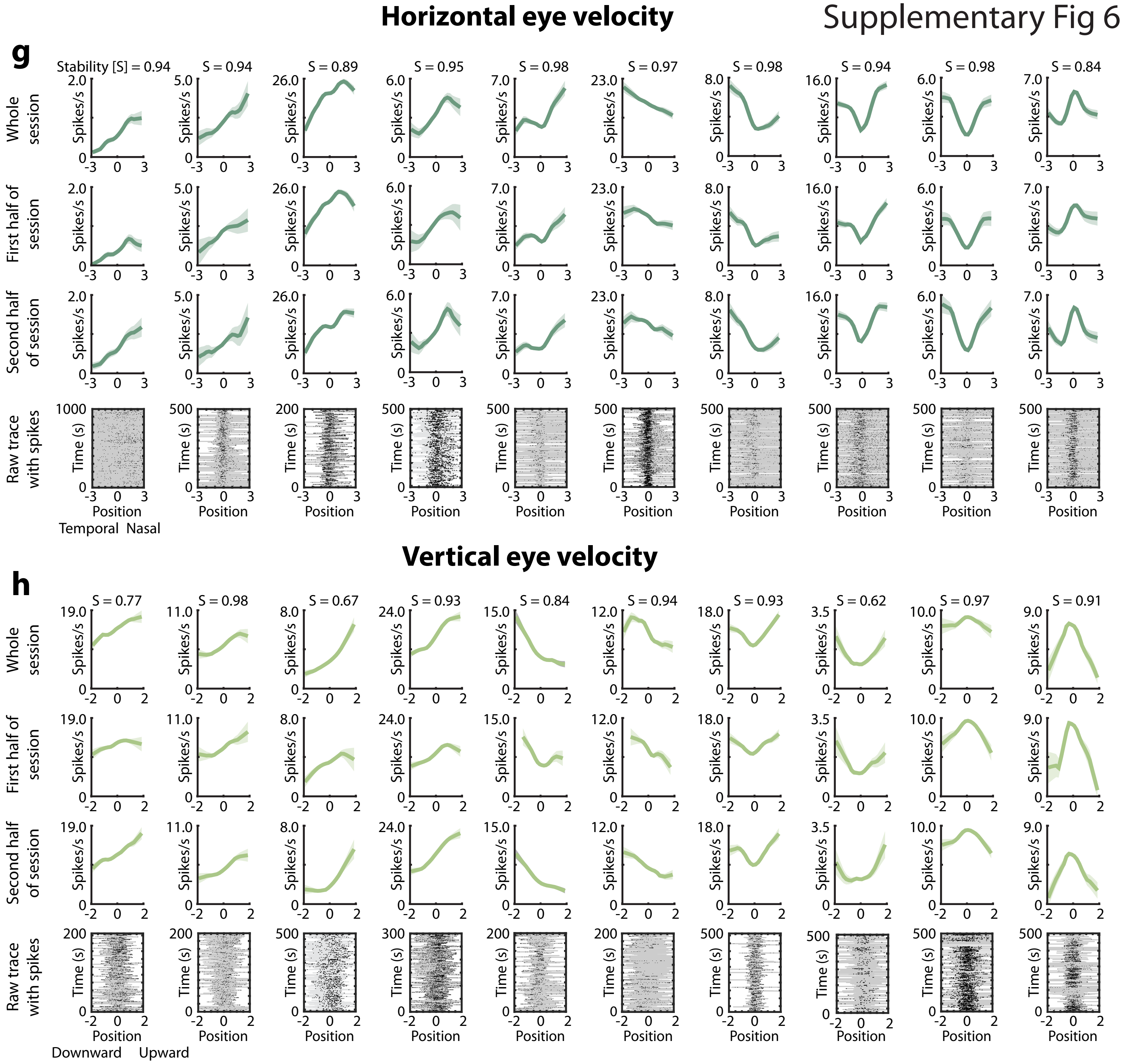
Horizontal eye velocity
g
Stability [S] = 0.94
S = 0.94
S = 0.89
S = 0.95
S = 0.98
S = 0.97
S = 0.98
S = 0.94
S = 0.98
S = 0.84
Whole session
First half of session
Second half of session
Raw trace with spikes
Spikes/s
Time (s)
Position
Temporal
Nasal
Vertical eye velocity
h
S = 0.77
S = 0.98
S = 0.67
S = 0.93
S = 0.84
S = 0.94
S = 0.93
S = 0.62
S = 0.97
S = 0.91
Downward
Upward

Supplementary Figure 6. Examples of raw tuning curves and raw behavioral traces with spikes overlaid. **a**, Illustration of how raw tuning curves were generated, using a pitch-encoding example cell. The top plot (a_i) shows a clip of the pitch position with spikes overlaid. Note that it can be difficult to discern a cell's tuning by eye from its raw data trace (a_i), as the raw data trace is not normalized by occupancy. To generate a tuning curve, we discretized pitch, computed the number of spikes in each bin (a_{ii}), and divided this by the number of time points in each bin (a_{iii}). The resulting raw tuning curve (a_{vi}) shows the cell's tuning to pitch. **b-h**, Top rows: Examples of raw tuning curves (mean ± sem firing rate in each bin) for cells significantly encoding head pitch (b), head roll (c), azimuthal head velocity (d), horizontal eye position (e), vertical eye position (f), horizontal eye velocity (g), or vertical eye velocity (h). The visualized behavioral ranges were selected according to the typical occupancies observed across mice and sessions (Figure 1b and Figure 4f-i). Top-middle and bottom-middle rows: the corresponding raw tuning curves constructed from the first half and second half of the session, respectively. The stability of the raw tuning curves, defined as the Pearson's correlation coefficient between the tuning curves for the first and second half of the session, is shown at top ('S'). Bottom rows: raw data traces corresponding to each raw tuning curve example above. The gray trace shows behavioral position (x-axis) across time (y-axis), for a portion of the session. Black dots indicate spikes. The extremal values of the raw data trace are clipped in order to allow direct comparison to the tuning curves above.

Supplementary Fig 7

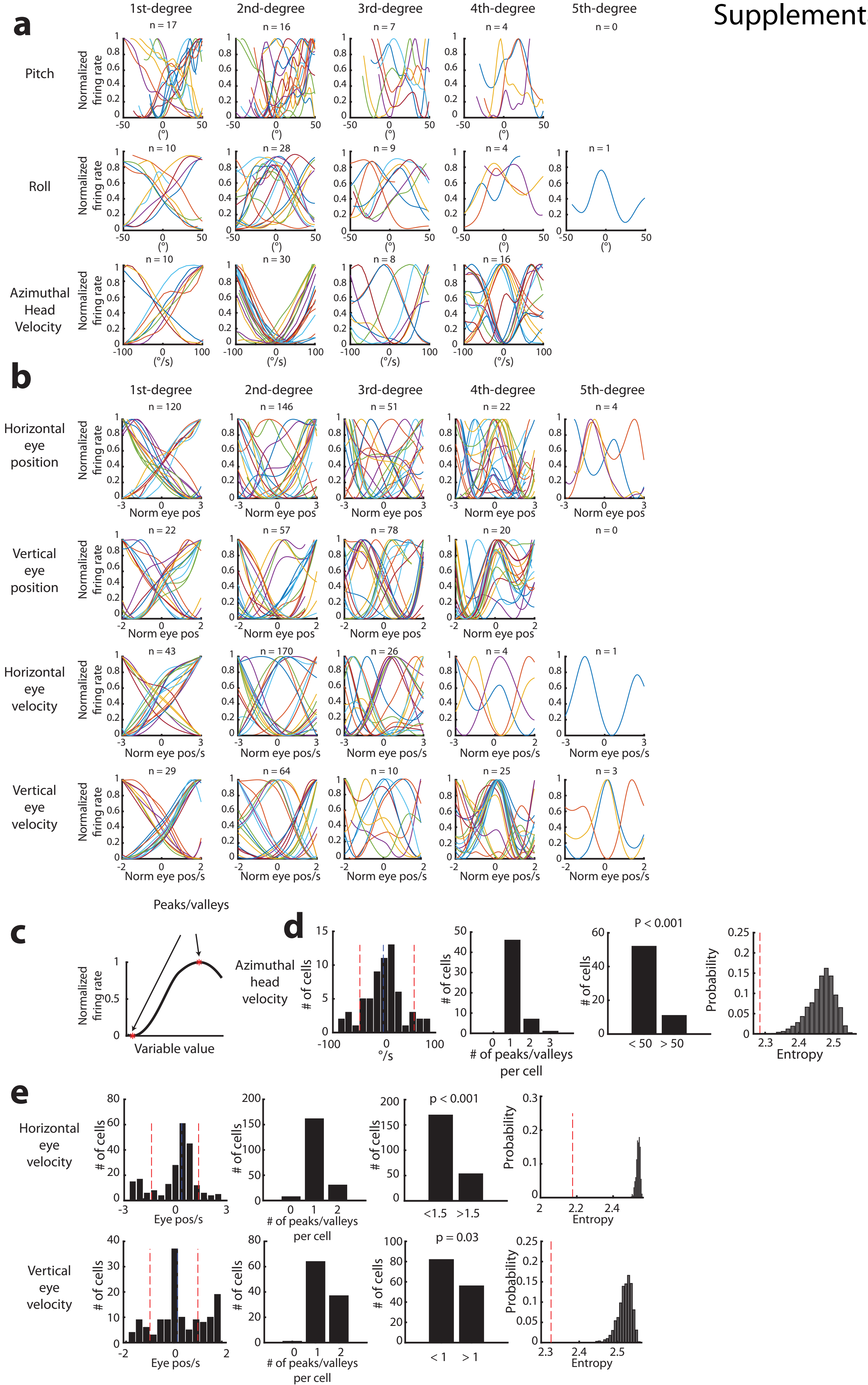

Supplementary Figure 7: Analysis of tuning curves using polynomial-based classification. **a-b,** Example normalized model-derived tuning curves. Tuning curves for pitch and roll were smoothed with a Gaussian filter ('smoothdata' MATLAB function, width = 20 bins). **c**, Example peak and valley detected in a tuning curve. **d**, Distribution of peaks/valleys in the azimuthal head velocity tuning curves are centered on 0 degrees/sec. left: The distribution of peaks/valleys detected across nonlinear cells encoding azimuthal head velocity. Blue dotted line indicates median value (not different from 0, $Z = 1.52$, $P = 0.13$, two-sided Wilcoxon signed rank test). Red dotted lines indicate the halfway value between 0 and the maximum/minimum azimuthal velocity. left-middle: Distribution of the number of peaks/valleys. right-middle: Number of peaks/valleys with absolute azimuthal velocity < 50 degrees/second exceeded the number of peaks/valleys with absolute azimuthal velocity > 50 degrees/second (n = 52/63, binomial test, Z = 5.04, $P = 2.0\text{e-}7$). right: The entropy of the distribution of peak/valleys is lower than null entropy distribution under a uniform distribution (with $P = 0.0020$). **e**, Distribution of peaks/valleys for horizontal eye velocity (top) and vertical eye velocity (bottom) tuning curves are centered on 0 eye position units/sec. left: Distribution of peaks/valleys detected across nonlinear cells encoding horizontal (top) or vertical (bottom) eye velocity. Blue dotted line indicates the median value (horizontal: different from 0, $Z = 2.63$, $P = 0.0080$, vertical: different from 0, $Z = 3.34$, $P = 8.0\text{e-}4$; two-sided Wilcoxon signed rank test). Red dotted lines indicate halfway value between 0 and the maximum/minimum horizontal or vertical velocity. left-middle: Distribution of the number of peaks/valleys. right-middle: The number of peaks/valleys with an absolute velocity within red dotted lines compared to the number of peaks/valleys outside the red dotted lines (horizontal n = 170/224, binomial $Z = 7.68$, $P = 1.0\text{e-}8$; vertical n = 82/138, binomial $Z = 2.13$, $P = 0.03$). right: The entropy of the distribution of peak/valleys for both variables is lower than null entropy distribution under a uniform distribution (both $P = 1.0\text{e-}4$).

Supplementary Fig 8

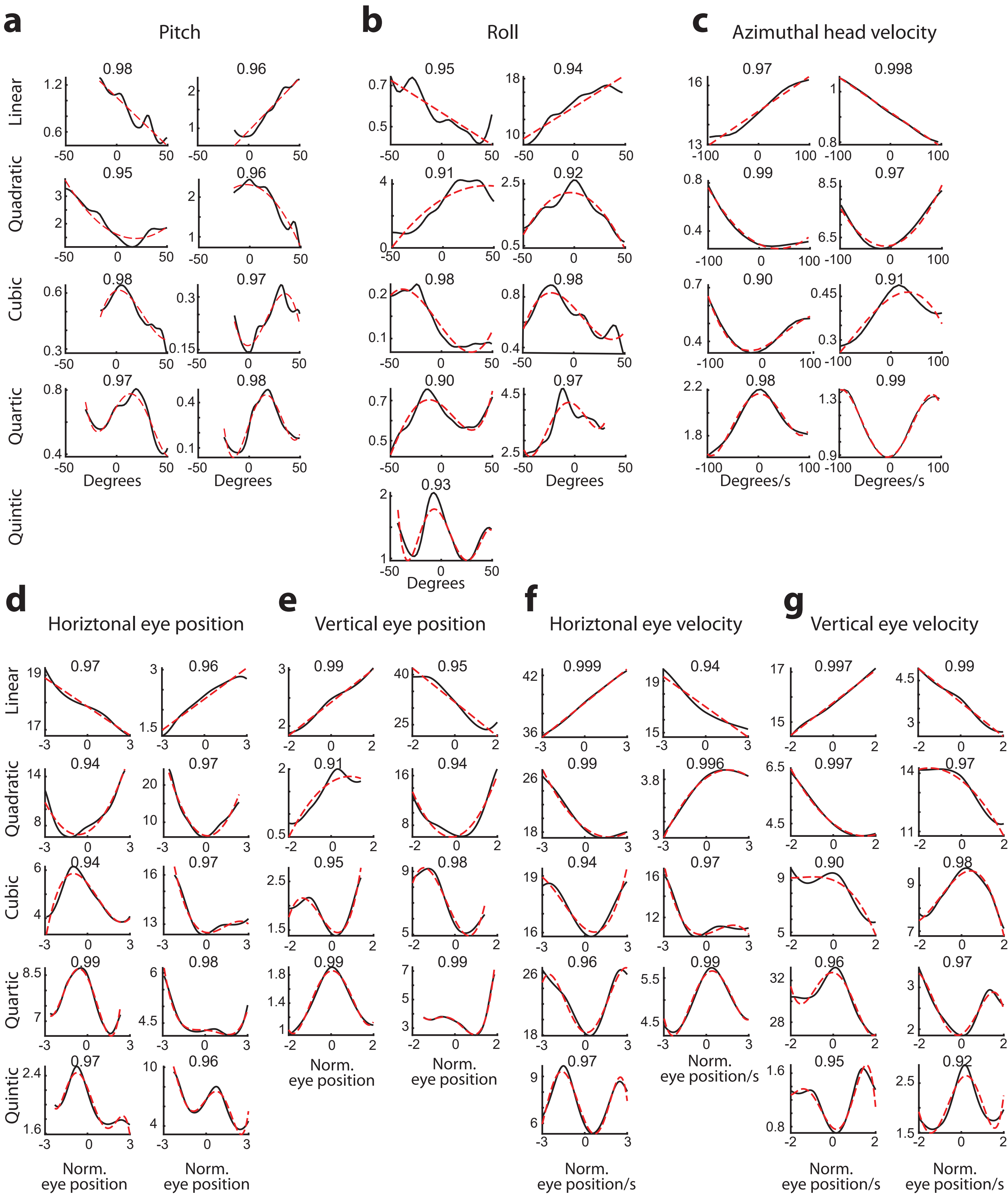

Supplementary Figure 8: Example model-derived tuning curves with polynomial fit overlaid. Each panel (a-g) gives examples of model-derived tuning curves for cells tuned to pitch (a), roll (b), azimuthal head velocity (c), horizontal eye position (d), vertical eye position (e), horizontal eye velocity (f) or vertical eye velocity (g). Rows in each panel correpond to the selected polynomial; the first row gives examples of cells where the linear fit resulted in >90% variance explained, the second row examples of cells where the quadratic fit resulted in >90% variance explained, and so on. Empty rows indicate that no cells of that type were identified (e.g. there were no cells with a 5th order polynomial fit for pitch). Black lines correspond to the model-derived tuning curve, while red dashed lined correspond to the polynomial fit. The number in the title of each graph indicates the variance explained by the polynomial.

Supplementary Fig 9

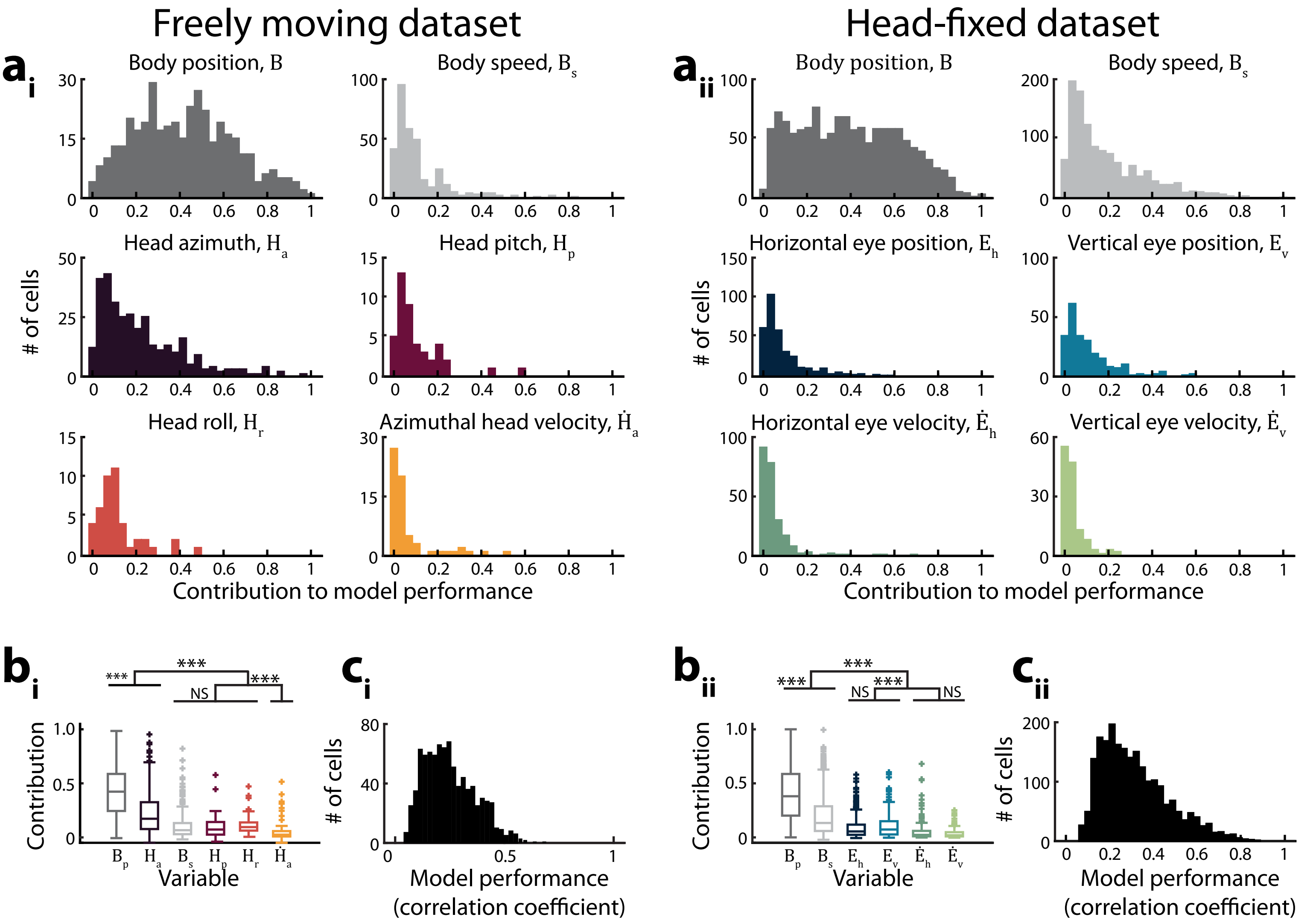

Supplementary Figure 9: Quantifying the contribution of each variable to model fit using correlation coefficients. We report the contribution of each variable to the correlation coefficient between a cell's smoothed spiketrain and the model-predicted firing rate. **ai-ci**, freely moving dataset, **aii-cii**, head-fixed dataset. **ai**, The normalized contribution of each behavioral variable to the correlation coefficient (median [1st–3rd quartile], n = # of cells encoding variable of interest and at least one other variable; B = 0.42 [0.24–0.59], n = 394; H_a = 0.17 [0.08–0.33], n = 327; B_s = 0.07 [0.03–0.13], n = 332; H_p = 0.07 [0.03–0.14], n = 44; H_r = 0.10 [0.06–0.14], n = 44; $\dot{H}_a$ = 0.02 [0.01–0.06], n = 64). **bi**, Comparison of each variable's contribution (# of cells as in (ai); X^2 = 488.5, P = 2.4e-103, df = 5; Kruskal-Wallis test followed by two-sided *post hoc* Wilcoxon rank-sum comparisons with α = 0.0033 after Bonferroni correction for multiple comparisons). ***for all significant pairwise comparisons, P < 0.0017. **ci**, Correlation coefficient for all cells significantly encoding at least one variable (median [1st–3rd quartile]; 0.23 [0.16–0.33]; n = 869). **aii**, The normalized contribution of each variable to the correlation coefficient (median [1st–3rd quartile], n = # of cells encoding the variable of interest and at least one other variable; B = 0.38 [0.20–0.59], n = 1319; B_s = 0.13 [0.06–0.29], n = 1187; E_h = 0.06 [0.02–0.12], n = 335; E_v = 0.07 [0.03–0.15], 231; $\dot{E}_h$ = 0.02 [0.01–0.06], n = 237; $\dot{E}_v$ = 0.02 [0.01–0.05], n = 132). **bii**, Comparison of model-contribution from each variable (# of cells as in (aii); X^2 = 1.4e3, P = 5.4e-292, df = 5; Kruskal-Wallis test followed by *post hoc* two-sided Wilcoxon rank-sum comparisons with α = 0.0033 after Bonferroni correction for multiple comparisons). ***for all significant pairwise comparisons, P < 2.9e-12. **cii**, Correlation coefficient for cells encoding at least one variable (median [1st–3rd quartile]; 0.29 [0.20–0.41]; n = 2191). Boxplots: box, interquartile range; solid line, median; whiskers, range; outliers plotted separately.

Supplementary Fig 10

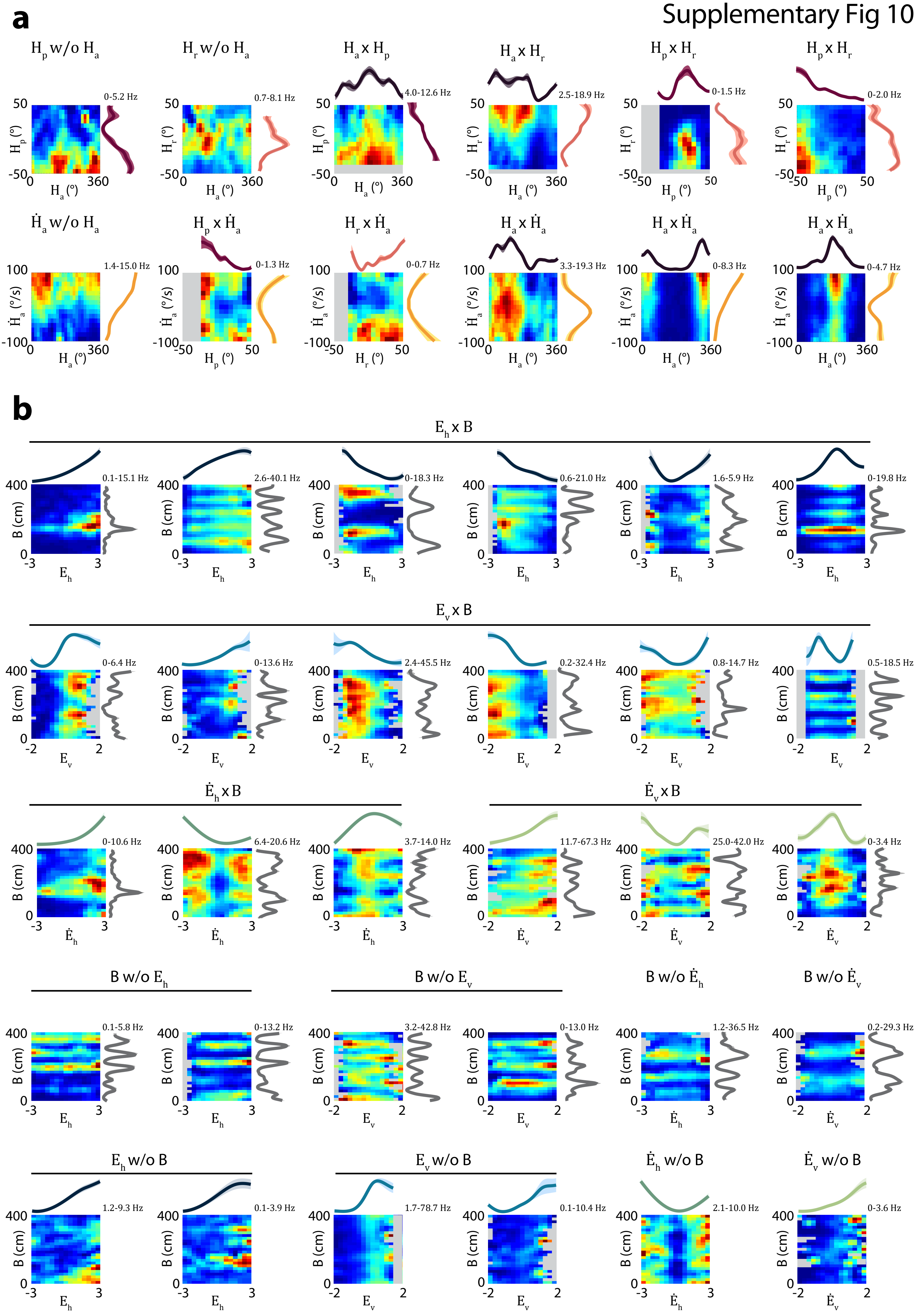

Supplementary Figure 10. Additional examples of joint tuning to multiple (**a**) head movement variables or (**b**) eye movement variables. Joint tuning curves were computed by binning each of the two variables within the ranges displayed and finding the average firing rate in each joint-bin. Examples where the cell encoded variable *a* but not *b* are labeled "*a* w/o b" (w/o; without). Examples where the cell encoded both variables *a* and *b* are labeled *"a x b".* The minimum and maximum firing rates are shown at top, with higher firing rates shown in warmer colors. Unvisited bins are shown in gray. The model-derived tuning curve for each significantly-encoded variable is shown beside the joint tuning curve. B, body position; B_s, body speed; H_a, azimuthal head direction; H_p, pitch; H_r, roll; $\dot{H}_a$, azimuthal head velocity; E_h, horizontal eye position; E_v, vertical eye position; $\dot{E}_h$, horizontal eye velocity; $\dot{E}_v$, vertical eye velocity.

Supplementary Fig 11

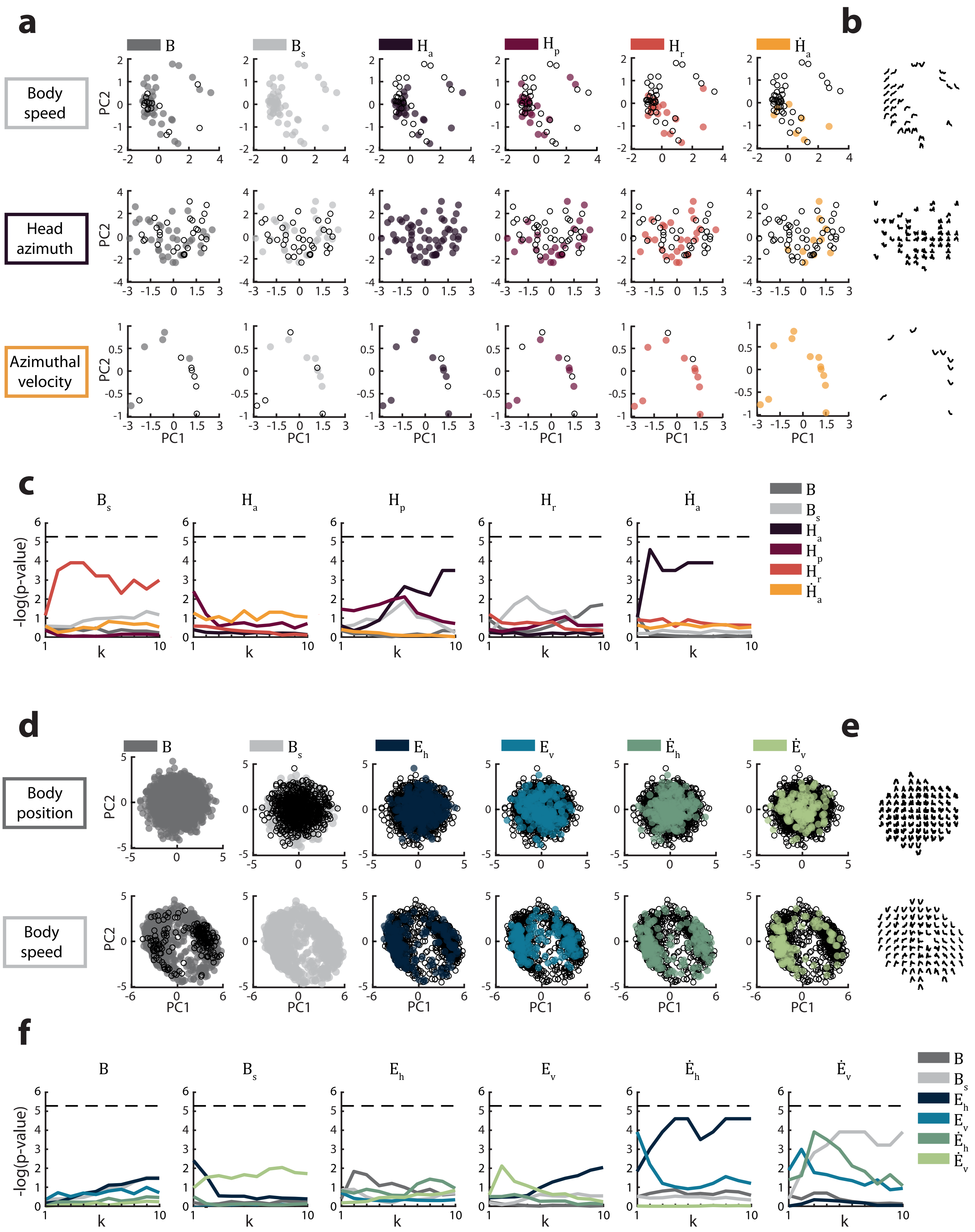

Supplementary Figure 11. Additional PCA clustering. **a**, Figure 3e shows the model-derived tuning curves of pitch and roll projected onto a two-dimensional 'tuning curve profile space'. Shown here are analogous plots constructed for cells encoding body speed, azimuth head direction, and azimuthal head velocity. Within a plot, each circle represents a cell whose tuning curves were projected onto the first two principal components. Subplots are colored according to whether or not the cell encoded each additional variable. **b**, Tuning curves from a subset of cells are shown at the cells' position in the tuning curve profile space. **c**, Results of the clustering statistical tests for the plots shown in (a) and in Figure 3e. These tests determine whether the tuning curves for a given variable tend to be more similar if the cells also encode another shared variable. The distance between the projection of cells' tuning curve determines tuning curve similarity. As one example, the first plot in (a) shows body speed-encoding cells projected in tuning curve space, colored according to whether each cell also encoded body position. The clustering test asks whether speed cells that also encode body position cluster together in this space more tightly than would be expected by chance. The primary behavioral variable is denoted at the top of each plot. The p-value for the statistical test at each value of k (number of nearest neighbors considered; 1:10) is shown, colored according to the secondary variable. The dotted line denotes the –log (base e) of the Bonferroni-corrected significance value of P = 0.05/10. **d**, As in (a) but for eye movement-related variables. Plots are analogous to those in Figure 7e, but constructed for cells encoding body position and body speed. **e**, Tuning curves from a subset of cells are shown at the cells' position in the tuning curve profile space. **f**, Results of the clustering statistical tests for the plots in (d) and Figure 7e. B, body position; B_s, body speed; H_a, azimuth head direction; H_p, head pitch; H_r, head roll; E_h, horizontal eye position; E_v, vertical eye position; $\dot{E_h}$, horizontal eye velocity; $\dot{E_v}$, vertical eye velocity.

Supplementary Fig 12

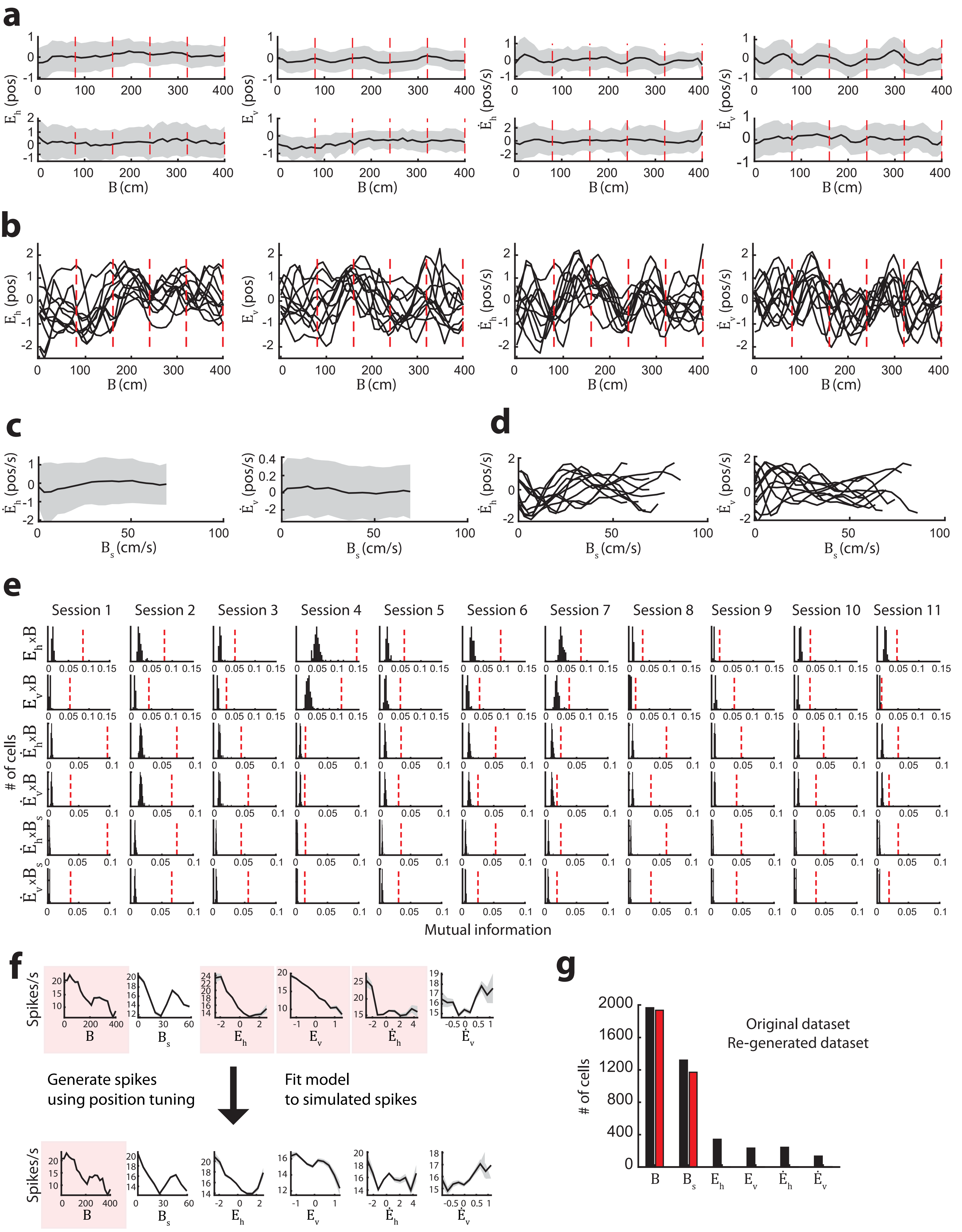

Supplementary Figure 12. Relationship between eye position/velocity and body position/speed. **a,** Representative data for the average horizontal and vertical eye positions, and the horizontal and vertical eye velocities, as a function of the position. Each row denotes a separate session. The black line indicates the mean, and the gray indicates standard deviation. Red lines denote the location of virtual landmarks. **b,** The horizontal and vertical eye position, and horizontal and vertical eye velocity, as a function of the position for all 11 sessions. Black lines indicate mean over each session. Red dotted lines denote the location of virtual landmarks. **c,** Representative data for the horizontal and vertical eye velocity as a function of the body speed for a single session. The black line indicates the mean, and the gray area indicates standard deviation. **d,** The horizontal and vertical eye velocity as a function of the animal's body speed for 11 sessions. Black lines indicate the mean over sessions. **e,** The mutual information across all sessions (columns) for horizontal eye position and body position (row 1), vertical eye position and body position (row 2), horizontal eye speed and body position (row 3), vertical eye speed and body position (row 4), horizontal eye speed and body speed (row 5), vertical eye speed and body speed (row 5). The red line indicates the mutual information. The histograms represent the mutual information computed from 100 iterations of shuffled data. Note that the data-derived mutual information is much higher than the shuffle-derived mutual information, indicating a statistically significant relationship between eye variables and body position/speed. **f,** A schematic describing the method used to verify whether the LN model separates eye-related information from body position/speed. The top row shows the tuning curves for each variable, and the model-selected encoded variables in pink, for actual data. The bottom row shows the same, but for data generated after 'deleting' eye-related contributions to spiking (see Methods). **g,** A bar graph indicating the variables encoded in the original dataset (black) and the generated dataset without eye information (red). Note that eye variables were not falsely detected.

Supplementary References

1. M. O. Pasquet *et al.*, Wireless inertial measurement of head kinematics in freely-moving rats. *Sci Rep* **6**, 35689 (2016).
2. J. J. Wilson, N. Alexandre, C. Trentin, M. Tripodi, Three-Dimensional Representation of Motor Space in the Mouse Superior Colliculus. *Curr Biol* **28**, 1744-1755 e1712 (2018).
3. A. F. Meyer, J. Poort, J. O'Keefe, M. Sahani, J. F. Linden, A Head-Mounted Camera System Integrates Detailed Behavioral Monitoring with Multichannel Electrophysiology in Freely Moving Mice. *Neuron* **100**, 46-60 e47 (2018).
4. F. Sargolini *et al.*, Conjunctive representation of position, direction, and velocity in entorhinal cortex. *Science* **312**, 758-762 (2006).
5. J. S. Taube, R. U. Muller, J. B. J. Ranck, Head-direction cells recorded from the postsubiculum in freely moving rats. I. Description and quantitative analysis. *J Neurosci* **10**, 420-435 (1990).
