# Supplementary material for: Mouse entorhinal cortex encodes a diverse repertoire of self-motion signals
Source: Nat Commun. 2021 Jan 28;12:671. doi: 10.1038/s41467-021-20936-8 (PMC7844029; doi:10.1038/s41467-021-20936-8)
Supplement: Supplementary file 3 — Reporting Summary [file 41467_2021_20936_MOESM3_ESM.pdf]

nature research

Corresponding author(s): Lisa M Giocomo

Last updated by author(s): Dec 18, 2020

Reporting Summary

Nature Research wishes to improve the reproducibility of the work that we publish. This form provides structure for consistency and transparency in reporting. For further information on Nature Research policies, see Authors & Referees and the Editorial Policy Checklist .

Statistics

For all statistical analyses, confirm that the following items are present in the figure legend, table legend, main text, or Methods section.

n/a	Confirmed	
☐	☒	The exact sample size (*n*) for each experimental group/condition, given as a discrete number and unit of measurement
☐	☒	A statement on whether measurements were taken from distinct samples or whether the same sample was measured repeatedly
☐	☒	The statistical test(s) used AND whether they are one- or two-sided<br>*Only common tests should be described solely by name; describe more complex techniques in the Methods section.*
☐	☒	A description of all covariates tested
☐	☒	A description of any assumptions or corrections, such as tests of normality and adjustment for multiple comparisons
☐	☒	A full description of the statistical parameters including central tendency (e.g. means) or other basic estimates (e.g. regression coefficient) AND variation (e.g. standard deviation) or associated estimates of uncertainty (e.g. confidence intervals)
☐	☒	For null hypothesis testing, the test statistic (e.g. *F*, *t*, *r*) with confidence intervals, effect sizes, degrees of freedom and *P* value noted<br>*Give P values as exact values whenever suitable.*
☒	☐	For Bayesian analysis, information on the choice of priors and Markov chain Monte Carlo settings
☒	☐	For hierarchical and complex designs, identification of the appropriate level for tests and full reporting of outcomes
☐	☒	Estimates of effect sizes (e.g. Cohen's *d*, Pearson's *r*), indicating how they were calculated

Our web collection on statistics for biologists contains articles on many of the points above.

Software and code

Policy information about availability of computer code

Data collection	Electrophysiological tetrode recordings were made using Axona Ltd systems (Axona Ltd., St Albans U.K., version 1.3.0.19, www.axona.com). Neuropixels recordings were made using SpikeGLX (version 20190214, billkarsh.github.io/SpikeGLX).
Data analysis	Spike sorting of Neuropixels recordings was performed using Kilosort2 (https://github.com/MouseLand/Kilosort2). Clusters were manually inspected and curated in Phy (https://github.com/cortex-lab/phy). Spike times and cluster identities were extracted from the output of KiloSort2 and Phy (version 2.0) using code from the Spikes repository (https://github.com/cortex-lab/spikes). Spike sorting for tetrode recordings was performed using Axona's Tint cluster-cutting software (version 2.4.6, www.axona.com). The built-in Matlab functions findpeaks, smoothdata, gausswin, fminunc and rlowess were used in data analysis (all Matlab version R2018a). All other analyses were conducted using custom code written in Matlab.

For manuscripts utilizing custom algorithms or software that are central to the research but not yet described in published literature, software must be made available to editors/reviewers. We strongly encourage code deposition in a community repository (e.g. GitHub). See the Nature Research guidelines for submitting code & software for further information.

Data

Policy information about availability of data

All manuscripts must include a data availability statement. This statement should provide the following information, where applicable:

- Accession codes, unique identifiers, or web links for publicly available datasets
- A list of figures that have associated raw data
- A description of any restrictions on data availability

The raw data used in all analyses within this manuscript are available at https://figshare.com/authors/Lisa_Giocomo/9864194.

Field-specific reporting

Please select the one below that is the best fit for your research. If you are not sure, read the appropriate sections before making your selection.

☒ Life sciences ☐ Behavioural & social sciences ☐ Ecological, evolutionary & environmental sciences

For a reference copy of the document with all sections, see nature.com/documents/nr-reporting-summary-flat.pdf

Life sciences study design

All studies must disclose on these points even when the disclosure is negative.

Sample size	Sample sizes were based upon on convention in the field (e.g., Alexander et al., (2020), Egocentric boundary vector tuning of the retrosplenial cortex, and Hardcastle, Ganguli, Giocomo (2015), Environmental boundaries as an error correction mechanism for grid cells). For open-field, tetrode recordings, data came from at least 6 animals. For Neuropixels probes recordings, data came from 8 animals.
Data exclusions	For open field recordings, sessions with poor coverage of the environment were excluded (Methods section 1.5, "Position estimate and cell type classification"). Further behavioral coverage criterion were applied in some analyses (Methods section 3, "Quantifying tuning curve shape"). These exclusion criterion were based on convention in the field (e.g., Giocomo et al., (2011), Grid cells use HCN1 channels for spatial scaling) and were pre-established.
Replication	The novel coding for head and eye movement-related variables was identified in the majority of individual animals (pitch-encoding cells found in 6/6 mice; roll-encoding cells found in 6/6 mice; azimuthal head velocity cells found in 18/25 mice; horizontal eye position-encoding cells found in 8/8 mice; vertical eye position-encoding cells found in 7/8 mice; horizontal eye velocity-encoding cells found in 8/8 mice; vertical eye velocity-encoding cells found in 7/8 mice).
Randomization	Randomization is not applicable to this study, as all findings are presented for wildtype animals. Animals within a given experimental group (i.e., open-field tetrode recordings with intertial measurement units, or head-fixed neuropixels recordings) were subjected to the same behavioral and recording protocols. To account for potential behavioral covariates (such as running speed), we included these variables within the linear non-linear Poisson model; the impact of such variables on entorhinal spiking, as well as interactions with newly-identified self motion signals, are described within the manuscript.
Blinding	Blinding was not applicable to this study, as all findings are presented for wildtype animals and there were no experimental groups.

Reporting for specific materials, systems and methods

We require information from authors about some types of materials, experimental systems and methods used in many studies. Here, indicate whether each material, system or method listed is relevant to your study. If you are not sure if a list item applies to your research, read the appropriate section before selecting a response.

Materials & experimental systems

n/a	Involved in the study
☒	☐ Antibodies
☒	☐ Eukaryotic cell lines
☒	☐ Palaeontology
☐	☒ Animals and other organisms
☒	☐ Human research participants
☒	☐ Clinical data

Methods

n/a	Involved in the study
☒	☐ ChIP-seq
☒	☐ Flow cytometry
☒	☐ MRI-based neuroimaging

Animals and other organisms

Policy information about studies involving animals; ARRIVE guidelines recommended for reporting animal research

Laboratory animals	The majority of the data came from male and female C57BL/6J mice, aged 2-12 months (n = 22). Additional data came from male and female hybrid C57BL/6J:129SVEV mice, aged 2-12 months (n = 12). The exact numbers of animals used are reported in Methods section 1.1, 'Animals'.
Wild animals	The study did not involve wild animals.
Field-collected samples	This study did not involve field-collected animals.
Ethics oversight	All procedures were approved by Stanford University's Administrative Panel on Laboratory animal Care (Methods section 1.1, 'Animals')

Note that full information on the approval of the study protocol must also be provided in the manuscript.
