# Supplementary material for: Mouse entorhinal cortex encodes a diverse repertoire of self-motion signals
Source: Nat Commun. 2021 Jan 28;12:671. doi: 10.1038/s41467-021-20936-8 (PMC7844029; doi:10.1038/s41467-021-20936-8)
Supplement: Supplementary file 2 — Peer Review File [file 41467_2021_20936_MOESM2_ESM.pdf]

Editorial Note: This manuscript has been previously reviewed at another journal that is not operating a transparent peer review scheme. The manuscript was considered suitable for publication without further review at Nature Communications.
